# Neutrophil‐Mediated Tumor‐Targeting Delivery System of Oncolytic Bacteria Combined with ICB for Melanoma Lung Metastasis Therapy

**DOI:** 10.1002/advs.202301835

**Published:** 2023-08-10

**Authors:** Lina Liu, Wenjie Xin, Qiang Li, Baolian Huang, Te Yin, Siqi Hua, Chen Yang, Chen Chen, Chao Han, Zichun Hua

**Affiliations:** ^1^ The State Key Laboratory of Pharmaceutical Biotechnology School of Life Sciences Nanjing University Nanjing Jiangsu 210023 China; ^2^ School of Biopharmacy China Pharmaceutical University Nanjing Jiangsu 210023 China; ^3^ Changzhou High‐Tech Research Institute of Nanjing University and Jiangsu TargetPharma Laboratories Inc. Changzhou Jiangsu 213164 China

**Keywords:** neutrophil, PD1 nanobody, tumor‐targeting delivery system, VNP20009

## Abstract

Oncolytic bacteria are the most promising tumor target vector. Questions also remain regarding finding a balance between the therapeutic efficacy and safety of oncolytic bacteria. The critical measure of how this balance is maintained is the improvement in tumor colonization. Attenuated *Salmonella typhimurium* (VNP20009) as the only *Salmonella* strain to be evaluated in a clinical trial is a potential tumor therapeutic bacterium. A delivery system with controlled release of VNP after being loaded into neutrophils, which significantly increases the tumor‐targeting of VNP and enhances its therapeutic efficacy in a melanoma lung metastasis model is constructed. To improve the synergistic therapeutic effect, a PD1 nanobody is applied to this system (NE(PD1nb)). NE(PD1nb) activate dendritic cells (DCs) differentiation and stimulate the M1‐like differentiation of macrophages, and induce CD4^+^ T‐cells maturity and cytotoxic CD8^+^ T‐cells activation through DCs tumor antigen presentation.

## Graphical abstract

Oncolytic bacteria delivery system based on neutrophil camouflage is developed to increase tumor colonization and safety. VNP‐PD1nb are loaded by neutrophils in vitro; NE(PD1nb) are transported along the blood vessels after intravenous injection and colonize in lung tumor foci. NE(PD1nb) induce antitumor immune response, thus, achieving consolidated therapy with bacteriotherapy, cell‐based immunotherapy, and immune checkpoint blockers immunotherapy.

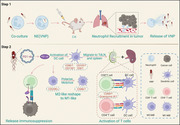

## 1 Introduction

In the original report of using oncolytic bacteria from 1813, Vautier observed that patients recover from tumors owing to developing gas gangrene.^[^ ^1^ ^]^ Then, Coley's toxin, which is composed of live *Streptococcus pyogenes*, was created by William B. Coley and used for cancer treatment and even cured many patients with this disease.^[^ ^2^, ^3^, ^4^ ^]^ Based on these reports, the field of bacterial cancer therapy has been widely valued. To date, numerous oncolytic bacterial strains have been explored, including *Salmonella*, *Listeria*, *Escherichia*, etc.^[^ ^2^, ^5^ ^]^ Attenuated *Salmonella typhimurium* VNP20009 (VNP), in which the *purI* and *msbB* genes were deleted to reduce side effects and the potential of septic shock, is one of the most promising strains.^[^ ^4^, ^6^, ^7^ ^]^ Compared with wild‐type *Salmonella typhimurium*, VNP has a remarkable tumor enrichment ability and a certain degree of side effects in a mouse tumor model. The titer of VNP in tumors is ≈1000 times higher than that in other organs.^[^ ^8^ ^]^

In addition, VNP, as the only *Salmonella* strain that has been evaluated in clinical trials, is a potential tumor therapeutic bacterium. In a phase I clinical trial, most of the patients with metastatic melanoma had no significant side effects after treatment with VNP, except at the highest dose. However, only in the highest dose group was VNP colonization in tumor tissue observed.^[^ ^9^ ^]^ The colonization ratio of oncolytic bacteria is the most vital factor in tumor therapy. Questions also remain regarding finding a balance between the therapeutic efficacy and safety of oncolytic bacteria. Many excellent studies have reported on ways to enhance the therapeutic efficacy of *Salmonella*, such as by carrying shRNA against a critical gene of metastasis^[^ ^10^, ^11^, ^12^ ^]^ or upregulating tumor genes,^[^ ^13^ ^]^ expressing effector proteins, such as IFN‐γ,^[^ ^14^, ^15^ ^]^ cytotoxic proteins,^[^ ^16^ ^]^ tumor‐specific antigens,^[^ ^17^ ^]^ covalently attaching photosensitizers,^[^ ^18^, ^19^, ^20^ ^]^ or cytotoxic biomaterials.^[^ ^21^ ^]^ These works were devoted to enhancing the tumor‐killing ability of oncolytic bacteria; however, they failed to address tumor colonization.

Recent studies have shed new light on the systemic wrapping as a feasible measure to increase tumor colonization by oncolytic bacteria. A remarkably engineered strain was designed in which the surface was wrapped with capsular polysaccharide, a natural extracellular biopolymer that can transiently reduce the immunogenicity of oncolytic bacteria.^[^ ^22^ ^]^ The strategy successfully enhanced bacterial safety and therapeutic efficacy at the same time by transiently protecting the bacteria. Notably, *Salmonella* covalently attached to sialic acid‐decorated AuNPs could recognize L‐selectin on the surface of neutrophils, hence realizing the tumor‐homing colonization of oncolytic bacteria.^[^ ^23^ ^]^ There is a body of evidence indicating that VNP colonizes tumors by penetrating from the blood vessels into the tumor core,^[^ ^24^, ^25^ ^]^ which suggests that the rapid clearance of VNP from blood may result in poor tumor colonization. This hypothesis was confirmed by Westphal et al.’s work, which reported that the depletion of host neutrophils leads to an increased titer of bacteria in tumors.^[^ ^26^ ^]^ In addition, our previous work proved that tumor therapeutic efficacy was enhanced by depletion of host neutrophils.^[^ ^27^ ^]^ Accordingly, we assume that neutrophils are a key factor in VNP tumor targeting in vivo.

It is generally believed that neutrophils are the first line of defense in the innate immune system, as they phagocytose and capture pathogens.^[^ ^28^ ^]^ To date, as mature drug delivery system vectors, neutrophils load various types of drugs and target tumor cores.^[^ ^29^, ^30^, ^31^ ^]^ Neutrophils load drugs by phagocytosis and target tumor foci, subsequently releasing the drug by cell lysis.^[^ ^32^, ^33^, ^34^, ^35^, ^36^ ^]^ Given these data, we assumed that neutrophils can load VNP and transiently avoid attack from the immune system, hence increasing the colonization of VNP in the tumor core. Therefore, in this work, we confirmed that neutrophils are a critical factor in VNP colonization. In addition, we systematically addressed the balance between the safety and therapeutic efficacy of VNP by constructing a VNP delivery system in which neutrophils release VNP into the tumor core. To further enhance the therapeutic efficacy of the release system, an engineered strain secreting the PD1 nanobody was used. This modified system remodeled the tumor microenvironment (TME), which is characterized by a significant increase in activated dendritic cells (DCs), cytotoxic T cells, and antitumor macrophages.

## 2 Results

### 2.1 Neutrophils are a Key Factor in VNP Tumor Targeting In Vivo

**Figure 1:**
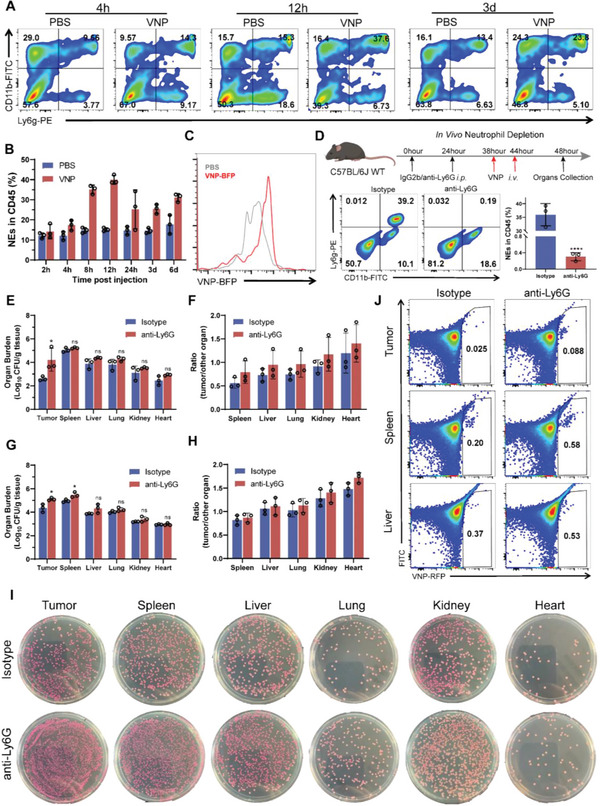
Neutrophil is a critical factor in VNP tumor targeting. A) After VNP injection (i.v.), the proportion of neutrophils in blood at different time points by FACS. B) The statistic graph of neutrophils proportion. C) The histogram of VNP‐BFP positive in neutrophils. D) The schema of neutrophils depletion in the tumor‐bearing mouse: the tumor‐bearing mouse was pretreated twice (i.p.) with anti‐Ly6g or IgG2b, and after 48 h, the ratio of neutrophils in blood by FACS was detected; 4 or 10 h after VNP administration, organ tissues were collected and homogenate to evaluate the organ burden of VNP. E) The organ burden of VNP 4 h after injection. F) The ratio of tumor to other organs after 4 h injection. G,H) The organ burden and the ratio of VNP 10 h after administration. I) 4 h after administration, tissues were homogenized and planted on LB agar after dilution. J) The proportions of VNP‐RFP in different tissues were measured by FACS after 4 h VNP injection. Data are shown as the mean ± SD. **** *p* < 0.0001, *** *p* < 0.001, ** *p* < 0.01, * *p* < 0.05, ns: no significance.

Bacteria are transported into the tumor core due to the permeability of blood vessels.^[^ ^37^ ^]^ Moreover, VNP was cleared from mouse blood within 12 h and from monkeys within 6 h.^[^ ^8^ ^]^ To explore the changes in the blood, blood was collected and the proportion of neutrophils was measured at different time points (2–144 h) after VNP administration (**Figure** **1**A,B). The results indicated that the proportions of neutrophils were significantly increased at 8 h and peaked at 12 h, which suggests that neutrophils were mobilized to clear VNP. As expected, neutrophils phagocytosed VNP in peripheral blood, as shown in Figure 1C. Hours after VNP injection, VNP‐blue fluorescent protein (BFP) fluorescence was observed in host neutrophils. To determine the influence of neutrophils on VNP tumor targeting, we depleted neutrophils with anti‐Ly6g and then intravenously (i.v.) injected 1 × 10^6^ VNP into tumor‐bearing mice as described in Figure 1D. 4 h after administration, the neutrophil‐depleted group (anti‐Ly6g) displayed more VNP colonization than the control group (isotype‐IgG2b) in all organs, especially the tumor (Figure 1E,F). Similar results were obtained at 10 h. Of note, the bacterial titers in the spleen were significantly enhanced in the neutrophil‐depleted group (Figure 1G,H). As shown in Figure 1I, total tissue homogenates were diluted and plated on Luria Bertani (LB) agar plates, and the numbers of bacterial colonies showed similar results to those above. In addition, we determined the ratio of VNP in the tissue homogenates by fluorescence‐activated cell sorting (FACS), and the results showed that the depletion of neutrophils significantly enhanced VNP colonization in the tumor, spleen, and liver (Figure 1J). Taken together, these data show that neutrophils decreased VNP tumor colonization by clearing VNP from the peripheral blood, and the depletion of neutrophils significantly enhanced VNP tumor targeting.

### 2.2 Neutrophils Load VNP and Maintain Their Activity, while VNP Releases and Leads to the Recovery of Proliferation

**Figure 2:**
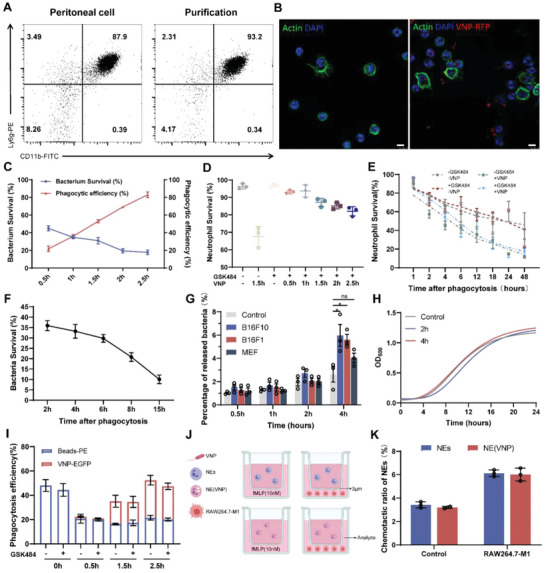
Peritoneal neutrophil can load VNP and release VNP after stimulation. A) The purity of peritoneal neutrophils was measured by FACS. B) The magnified fluorescent pictures of NE(VNP); 1.5 h after neutrophils incubated with VNP (MOI = 100:1); actin (green), 4′,6‐diamidino‐2‐phenylindole (DAPI, blue), VNP‐RFP (red). 8 µm scale bars shown. C) The efficiency of neutrophils‐loaded VNP and survival of VNP with different cocultured time points. D) The survival of neutrophils incubated with VNP. E) The survival of neutrophil after loading. F) The survival of intracellular VNP after loading. G) 1.5 h after neutrophil loading VNP, cocultured with B16F10, B16F1, MEF, or not and the supernatant was collected and planted on LB agar to count the percentage of intracellular VNP released. H) Released VNP were diluted according to scale and inoculated into LB to measure growth curves. I) Neutrophils were incubated with VNP (EGFP) at different times and then cocultured with the fluorescent beads (RFP) for 1 h, the efficiency of phagocytosis was detected by FACS. J) The experimental schema of the chemotactic assay: NEs and NE(VNP) were added to upper chambers (3 µm); the lower chambers were precoated with RAW(M1), or 10 × 10^−9^ m fMLP; after co‐incubating for different time points, the supernatants of lower chambers were collected to evaluate the number of neutrophils. K) The chemotactic proportion of neutrophils. Data are shown as the mean ± SD. **** *p* < 0.0001, *** *p* < 0.001, ** *p* < 0.01, * *p* < 0.05, ns: no significance.

After confirming that neutrophils are a critical factor in VNP tumor targeting, we postulated that neutrophils could load VNP to transiently escape immune system clearance. To validate this conjecture, we designed a VNP delivery system for neutrophil loading, which protects VNP from clearance by the blood and enhances VNP tumor targeting and biosafety. First, peritoneal neutrophils were harvested and purified by Percoll gradient centrifugation. The purity of the neutrophils was determined by FACS (**Figure** **2**A) and Giemsa staining (Figure S1A, Supporting Information). The results revealed that the extracted neutrophil subsets were more than 90% pure. Because neutrophils are fragile in vitro, they were incubated with GSK484, an inhibitor of PAD4,^[^ ^38^, ^39^ ^]^ to inhibit neutrophil death in vitro (Figure S1B, Supporting Information). As shown in Figure 2A and Figure S2A (Supporting Information), neutrophils can load VNP when in coculture. Moreover, transmission electron microscope was used to examine neutrophil loading of VNP (Figure S1C, Supporting Information). And the result reveals that the number of mitochondria was reduced in VNP‐loading neutrophil, and the phagosome occupied a larger volume. In addition, the nucleus of NE(VNP) exhibits an irregular shape with multiple lobes, which suggests that NE(VNP) is a mature state. The loading efficiency increased with incubation time (Figure S2B,C, Supporting Information). To evaluate the exact quantity of VNP loaded in each neutrophil, a regression line indicating the relationship between the mean fluorescence intensity (MFI) of VNP‐red fluorescent protein (RFP) and VNP number was plotted (Figure S2D, Supporting Information). As shown in Figure S2E (Supporting Information), 6.73 VNP were loaded per neutrophil after 1.5 h of incubation. In addition, neutrophils could load two kinds of VNP in a proportion of 1:1 (Figure S3A,B, Supporting Information).

To balance VNP activity and loading efficiency, the constructed biaxial plots indicated the percent bacterial survival and phagocytic efficiency (Figure 2C). We selected 1.5 h as the optimal time because at this point, the percent phagocytic efficiency reached 50% and bacterial survival reached 30%. Next, the activity of neutrophils at different time points was detected by AM/propidium iodide (PI) staining (Figure 2D). The results revealed that GSK484 significantly enhanced the survival of neutrophils, and the neutrophils maintained 85% survival after 1.5 h of incubation. Similarly, the survival of neutrophils 1.5 h after loading VNP was also measured (Figure 2E). The results indicated that at least 50% of the NE(VNP) activity was maintained after 6 h, at which time most intravenous neutrophils could target the tumor site. Based on these results, the activity of VNP was detected and found to change slightly in 6 h (Figure 2F), which gives intracellular VNP enough time to be released and target the tumor site. Next, to confirm that intracellular VNP could be released and recover its activity, after incubation with B16F1 cells and the more malignant tumor cells B16F10, the percentage of VNP released was measured with mouse embryonic fibroblasts (MEFs) as a control. As shown in Figure 2G, compared with the untreated control group, B16F10 cells induced a significant increase in VNP release, approximately twofold more than that released by B16F1 cells. To explore the efficiency of the recovery of released VNP, we collected the VNP released into the supernatant, planted it into LB, and then measured the growth curve (Figure 2H). To avoid the influence of bacterial number, different dilutions (100‐fold and 1000‐fold) were made, and these growth curves were found to be similar. These results imply that the intracellular release of VNP from neutrophils could recover the proliferative activity.

In addition, to determine whether neutrophils could maintain their antitumor ability after VNP loading, we evaluated the phagocytosis and chemotactic ability of NE(VNP). As shown in Figure 2I, the loaded VNP influenced neutrophil phagocytosis of fluorescent beads; however, the total phagocytosis efficacy (VNP and beads) was maintained (Figure S4A, Supporting Information). To further ensure the status of neutrophil, the “eat‐me” signal and “do not eat‐me” signal were measured by FACS and real‐time polymerase chain reaction (RT‐PCR). The previous works reported that Annexin I expressed on the plasma membrane has also been identified as an “eat me” signal. And Annexin I then colocalizes with PtdSer (PS) and effectively stimulates engulfment of the apoptotic cells. In addition, CD47 is expressed on the membrane surface of healthy cells and is recognized by the inhibitory receptor, signal regulatory protein alpha (SIRPα), to inhibit the engulfment by macrophages.^[^ ^40^ ^]^ The PS level was evaluated by Annexin V staining, and the result indicated that PS‐negative neutrophils after isolation maintain more than 90% (Figure S4B,C, Supporting Information), and the VNP‐loaded neutrophils could maintain more than 80%, which was consistent with the AM/PI staining result. And then, the expression level of “eat‐me” signal (*Annexin I*) and “do not eat‐me” signal (*CD47*) in neutrophils was measured by RT‐PCR. The result reveals that the slight upregulated expression of *Annexin I* and the significant upregulated expression of *CD47* were identified (Figure S4D, Supporting Information), which suggests that there may be a small population of neutrophil damage.

To further validate our notion, the neutrophils were labeled by DiI dye after loading VNP and co‐cultured with M0‐like and M1‐like RAW264.7 cells at 2 h. In NEs group, the percentage of DiI positive in F4/80^+^ cells was 8% and 11% after incubation with M0‐like and M1‐like RAW264.7 cells, respectively (Figure S4E, Supporting Information). And in the NE(VNP) group, the percentage was ≈12% and 14% (Figure S4F,G, Supporting Information). These results suggest that a small population of neutrophil was swallowed by macrophages, and this is inevitable. In general, most of neutrophils preserve their cellular integrity after culture and could migrate to tumor cores. In addition, neutrophils preserve their cellular integrity after culture, which is longer than other cell types, a phenomenon proposed to rely on to the use of glycolysis for energy production.^[^ ^41^ ^]^

In the chemotactic assay, 10 × 10^−9^ m *N*‐formylmethionyl‐leucyl‐phenylalanine (fMLP) or M1‐like type RAW264.7 cells were seeded into the lower chamber of a 3 µm Transwell chamber, and the upper chamber was seeded with NE(VNP) or untreated neutrophils (NEs) as controls. 2 h after incubation, the supernatant in the lower chambers was collected to count the cells (Figure 2J). As shown in Figure 2K, NE(VNP) had similar chemotactic ability compared with control NEs; in addition, B16F10 tumor cells induced more NEs chemotaxis.

### 2.3 NE(VNP) Polarized to the Antitumor Type (N1 Type) and Induced More Tumor Apoptosis

**Figure 3:**
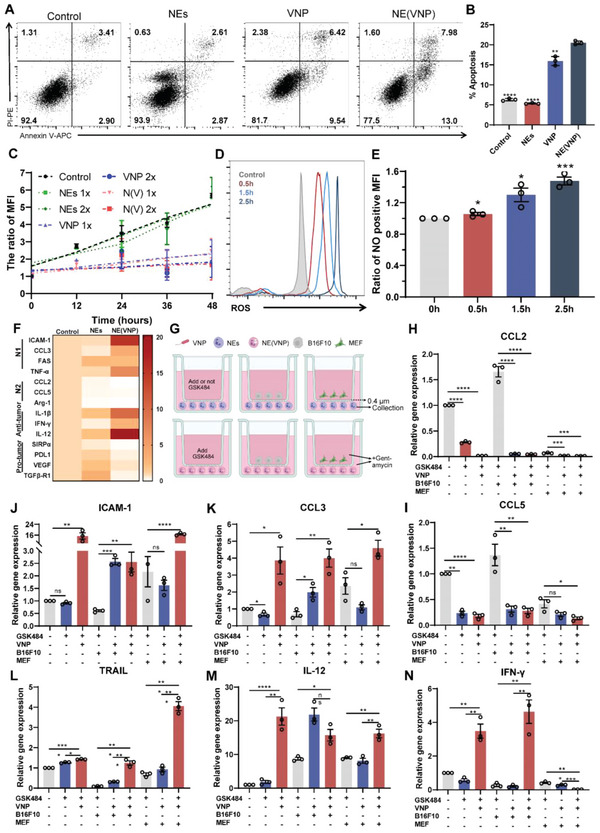
NE(VNP) showed antitumor phenotypes. A,B) The apoptosis levels of B16F10 after incubated with NEs, VNP, NE(VNP), or not for 16 h. C) The proliferation of B16F10‐GFP after incubation with NEs, VNP, NE(VNP), or not. D) The ROS level of neutrophils. E) The NO level of neutrophils. F) The gene expression levels of antitumor and pro‐tumor neutrophils were detected by real‐time PCR. G) The schema of co‐culture experiment: NEs or NE(VNP) were cultured with GSK484 or not for 1 h; and B16F10, MEF, or not on top of a 0.4 µm transwell membranes and NEs or NE(VNP) below the transwell insert for 4 h, and the cells in the lower chamber was collected to detect gene expression. In addition, 45 µg mL^−1^ gentamicin sulfate was maintained at all times. H,I) The mRNA expression levels of “N2” markers (*CCL2* and *CCL5*). J,K) The mRNA expression levels of “N1” markers (*ICAM‐1* and *CCL3*). L–N) The mRNA expression levels of antitumor cytokines (*TRAIL*, *IL‐12*, and *IFN‐γ*). Data are shown as the mean ± SD. **** *p* < 0.0001, *** *p* < 0.001, ** *p* < 0.01, * *p* < 0.05, ns: no significance.

Then, the influence of NE(VNP) on tumor cells was evaluated by Annexin V/PI and terminal deoxynucleotidyl transferase biotin‐dUTP nick end labeling (TUNEL) staining. As shown in **Figure** **3**A,B, 16 h after incubation, the apoptosis level of B16F10 cells in the NE(VNP) group (20%) was significantly higher than that in the VNP group (15%), which suggests that NE(VNP) killed more B16F10 cells. In addition, TUNEL staining was used to verify this conclusion (Figure S5B, Supporting Information). By the same token, the supernatants after 2 h of culture with NEs, VNP, and NE(VNP) were collected and cultured with B16F10 cells for 16 h, and the results revealed that the supernatant of the NE(VNP) stimulated tumor cell apoptosis (Figure S5C,D, Supporting Information). Moreover, NEs, VNP, and NE(VNP) were incubated with B16F10‐green fluorescent protein (GFP) cells for different lengths of time, and the proliferation of B16F10‐GFP cells was evaluated by determining the ratio of MFI (Figure 3C). The results indicated that NE(VNP) inhibited tumor proliferation.

To elucidate the inhibitory mechanisms, the state of NE(VNP) was investigated by the level of reactive oxygen species (ROS), NO, and gene expression. As shown in Figure 3D, the ROS levels in neutrophils increased with incubation time. The NO level was determined by 4‐amino‐5‐methylamino‐2',7'‐difluorofluorescein diacetate (DAF‐FM DA) staining 1.5 h after incubation with VNP, and the NO level in the neutrophils increased 1.3‐fold compared with that in the untreated control group (Figure 3E and Figure S6A,B, Supporting Information). Overall, the state of NE(VNP) had significantly changed; hence, 1.5 h after incubation, NE(VNP) were collected to detect the gene expression levels by RT‐PCR. As shown in Figure 3F, the expression levels of the N1 antitumor type markers (*ICAM‐1*, *CCL3*, *FAS*, and *TNF‐α*) in NE(VNP) were significantly increased by approximately tenfold compared with the untreated control group, and in contrast, the expression of N2 protumor type markers (*CCL2*, *CCL5*, and *Arg‐1*) was significantly downregulated. Notably, antitumor cytokines, such as *IL‐1β*, *IFN‐γ*, and *IL‐12*, were upregulated ≈15‐fold, whereas neutrophil‐related tumor immune checkpoints, such as *SIRPα* and *PDL1*, and protumor markers, such as *VEGF* and *TGFβ‐R1*, were downregulated significantly.^[^ ^42^, ^43^, ^44^, ^45^ ^]^

To confirm that tumor‐infiltrated NE(VNP) maintained antitumor ability, the upper chamber of a 0.4 µm Transwell chamber was seeded with B16F10 cells, MEFs, or no cells, and NEs or NE(VNP) were seeded into the lower chamber and collected after incubation for 1 h, as shown in Figure 3G. Analogously, VNP induced tumor‐stimulated N2 neutrophil polarization to the antitumor type, which was highlighted by the downregulation of protumor markers (Figure 3H,I and Figure S7A,B, Supporting Information) and upregulation of antitumor markers (Figure 3J–N and Figure S7C–E, Supporting Information).

### 2.4 NE(VNP) Target and are Located at the Tumor Site

In keeping with the discussion above, NE(VNP) have more potent tumor‐killing capacity in vitro; accordingly, the tumor targeting ability and therapeutic capacity of NE(VNP) need to be validated in vivo. To confirm that VNP can locate to the tumor site owing to neutrophil loading and delivery, DBCO‐Cy5.5‐labeled neutrophils and VNP‐RFP were i.v. injected into tumor‐bearing mice, and the tumors were cut into sections and scanned after excision (Figure S8A,B, Supporting Information). As shown in Figure S8A (Supporting Information), VNP and the mobilized neutrophils were blocked at the border of the solid tumor at 2 h; in contrast, in the NE(VNP) group, VNP could infiltrate into the tumor core. At 4 h, the VNP group and the NE(VNP) group both showed that VNP was located at the tumor core, whereas the numbers of tumor‐infiltrating neutrophils were significantly increased in the NE(VNP) group, which suggests that VNP was protected by neutrophils and infiltrated the tumor.

**Figure 4:**
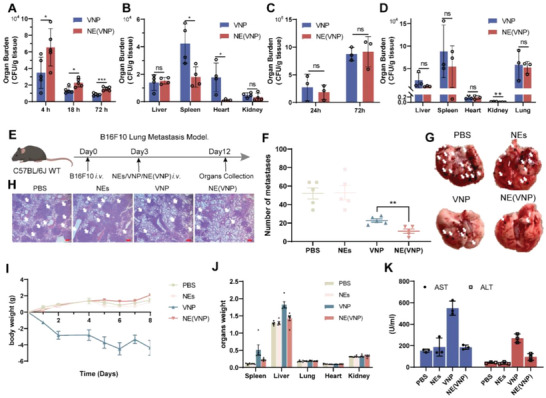
NE(VNP) enhanced tumor‐targeting and antitumor effect, and remised hepatosplenomegaly and body weight loss. A) 4, 18, and 72 h after VNP administration, the VNP titers of the lung tumor. B) The organ burden of VNP 3 days after administrations in the B16F10 lung metastasis model. C) 24 and 72 h after VNP administration, the tumor titers of VNP in the B16F10 subcutaneous xenograft model. D) 3 days after administrations, the organ burden of VNP in the B16F10 subcutaneous xenograft model. E) The schema of B16F10 lung metastasis experiment: 3 days after B16F10 injection, NEs, VNP, and NE(VNP) were administrated by i.v. and the tissues were collected at 12 days. F,G) The numbers and pictures of the lung metastasis foci. White arrows indicate metastasis foci (*n =* 5). H) The HE slices of lung 5 days after administration. White arrows indicate metastasis foci. 200 µm scale bars shown. I) The changing trend of body weight after administration. J) The organ weight of lung metastasis mouse. K) 5 days after injection, the levels of AST and ALT (*n =* 3). Data are shown as the mean ± SD. **** *p* < 0.0001, *** *p* < 0.001, ** *p* < 0.01, * *p* < 0.05, ns: no significance.

Neutrophils possess a tendency to naturally target chemotaxis to metastatic foci.^[^ ^46^ ^]^ To evaluate the tumor‐targeting ability of NE(VNP), a B16F10 lung metastasis model and a B16F10 subcutaneous xenograft model were established, and the tumors and organs were collected after excision to measure the VNP titer. As shown in **Figure** **4**A, compared with the VNP group, the titer of VNP increased twofold in the NE(VNP) group in the lung tumors. In addition, NE(VNP) lessened the clearance of VNP from the lung tumors. Consistent with this, the spleen burden and heart burden showed significant decreases in the NE(VNP) group compared with the VNP group, obviously decreasing by 1.5 times to two times (Figure 4B). To unambiguously identify the location of NE(VNP) at certain times, tumors were collected at different lengths of time after administration, and VNP‐RFP titers were measured by FACS (Figure S8E, Supporting Information). These results showed that VNP tumor colonization was observed in part of the tumor in NE(VNP) group at 2 h, by contrast, not in the VNP group (Figure S8F, Supporting Information). And at 12 and 36 h, both groups showed the colonization and proliferation of VNP in the tumor, however, the number of tumors infiltrating VNP was significantly increased in the NE(VNP) group, compared with the VNP group. As for 72 h, because of the limitation of tumor size and number, the killing of the host immune cells, and the growth inhibition of VNP, the titer of VNP maintained a steady trend. In the B16F10 xenograft model, the tumor burden of VNP was not different at different time points (Figure 4C); however, the titer of VNP in other organs decreased, such as in the liver, spleen, and kidney (Figure 4D). The tumor‐targeting ability of VNP was evaluated by counting the ratio of VNP titers between the tumor and other organs. NE(VNP) have a better tumor‐targeting ability than VNP (Figure S8F, Supporting Information). Overall, NE(VNP) were located in the tumor earlier than the time required by the VNP group, and more VNP were enriched in the tumor site than in other organs.

In this scenario, tissue distribution at 2 h was detected by in vivo imaging (Figure S8C,D, Supporting Information), and the results revealed that NE(VNP) were preferentially enriched in the lung, while VNP were enriched in the liver. Neutrophils have the ability to colonize and reside in the lung. Based on this, we postulated that the NE(VNP) delivery system has the potential to improve the difficulty with bacterial therapy in metastatic lung tumors.

Because neutrophils have a homing capacity to bone marrow, we detected the titers of VNP in bone marrow and metastatic lung tumors 12 h after administration in the lung metastasis model; this time point was selected based on the work reported by Luo et al. (Figure S9A, Supporting Information).^[^ ^47^ ^]^ The in vivo distribution result revealed that NE(VNP) had a better lung tumor targeting ability than VNP; in contrast, the bone marrow titer of NE(VNP) was slightly decreased compared with VNP. In addition, the ratio of the VNP burden in lung and bone marrow was increased significantly in the NE(VNP) group (Figure S9B, Supporting Information). These results suggest that VNP loaded into neutrophils did not increase their auto‐chemotactic ability to bone marrow. Previous research revealed that VNP could autotarget bone marrow in tumor‐free mice, and this phenomenon was observed in a monkey model. Some studies have indicated that the characteristics of bone marrow‐returned neutrophils are *CXCR4* upregulation and *CXCR2* deregulation. Based on this, the status of these genes in the NE(VNP) group was detected by RT‐PCR, and as expected, the gene expression level of *CXCR4* was decreased significantly (Figure S9C, Supporting Information). Moreover, neutrophils were cocultured with B16F10 cells, and gene expression was measured in a similar manner (Figure S9D–F, Supporting Information). In the NE(VNP) group, the expression of *CXCR4* was upregulated approximately twofold compared with that in the NE group; however, the expression of *CXCR2* was upregulated ≈14‐fold. The ratio of *CXCR2* to *CXCR4* relative gene expression increased (Figure S9F, Supporting Information); in other words, the marker of migrated out bone marrow had an advantage over that migrated in, which suggests that the NE(VNP) tended to stay in other tissues but not in bone marrow. The other neutrophil‐relative chemokine receptors, such as *CCR1*, *CCR5*, *CXCR1* in neutrophils were detected by RT‐PCR (Figure S9G, Supporting Information).^[^ ^48^, ^49^ ^]^ The result reveals that the expression level of *CCR1* and *CXCR1* was significantly upregulated after VNP loading, by contrast, there was no change in the expression of *CCR5*. The result suggests that neutrophils after being isolated had chemotactic potential.

### 2.5 NE(VNP) Enhanced the Safety of VNP and Inhibited B16F10 Lung Metastasis

According to the above observation, a B16F10 lung metastasis model was established and treated by i.v. injected NE(VNP), as shown in Figure 4E. In the NE(VNP) group, the number of B16F10 lung metastatic foci was significantly lowered compared with that in the phosphate‐buffered saline (PBS) group, and compared with that in the VNP group, a significant decrease in metastatic foci was also observed (Figure 4F–H). Bacterial therapy leads to weight loss and hepatosplenomegaly in tumor‐bearing mice losing weight after treatment, and both of these effects have been identified as side effects of bacterial therapy.^[^ ^50^ ^]^ Based on this, with the VNP delivery system NE(VNP), the mouse weight loss phenomenon disappeared entirely (Figure 4I). It is also noteworthy that the phenomenon of hepatosplenomegaly was significantly reduced (Figure 4J and Figure S10A,B, Supporting Information). Combining these data with those from hematoxylin and eosin (H&E) staining suggested that the phenomena of splenic and liver injuries were reduced, and other organs had no significant variations (Figure S10C, Supporting Information). Independent of this, the assessment of the biochemical function of the liver (AST and ALT) confirmed that all functional markers in the NE(VNP) group were reduced by ≈60% compared with those in the VNP group (Figure 4K). To evaluate the immunogenicity of NE(VNP), routine blood tests were performed 5 days after administration. All groups showed no abnormalities in the routine blood examination results, except for a decrease in platelets (PLTs) in the VNP group (Figure S11A–D, Supporting Information). To further detect the humoral immunity of NE(VNP), the serum total IgG levels were measured by enzyme‐linked immunosorbent assay (ELISA) in the tumor model mice (Figure S11E, Supporting Information) and normal mice (Figure S11F, Supporting Information). The results indicated that neutrophils and NE(VNP) have acceptable immunogenicity. However, comprehensive analysis of the body weight, organ weights, and biochemical indices of hepatic function and renal function guaranteed the safety of NE(VNP). Overall, the system of VNP delivery by NE(VNP) significantly enhanced the safety and therapeutic efficacy of VNP against B16F10 lung metastasis.

### 2.6 NEs Loaded with the Engineered Strain Showed Enhanced Therapeutic Efficacy against Lung Metastasis

To further improve the therapeutic efficacy of the NE(VNP) system, an engineered strain expressing the PD1 nanobody (PD1nb) was applied in this system. As Figure S12A (Supporting Information) shows, to ensure continuous PD1nb expression in vivo, an antiplasmid loss element and a strong constitutive promoter were applied for the expression plasmid, and pelB, a signal peptide, and a flag tag were fused with PD1nb to lead the nanobody to the periplasmic space in a hypoxic environment. Next, western blotting was used to evaluate the expression level of VNP‐PD1nb. The size of the flag‐fused nanobody was 17.8 kDa (Figure S12B, Supporting Information). Then, the growth curve of VNP‐PD1nb was constructed and the morphology was analyzed to ensure that the engineered strain could proliferate normally in vivo (Figure S12C,D, Supporting Information). As expected, VNP‐PD1nb had a similar morphology to VNP and proliferated normally.

**Figure 5:**
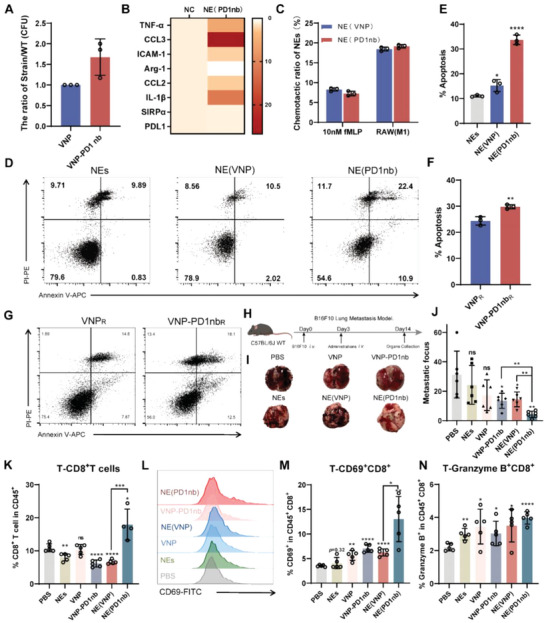
Neutrophil‐loaded engineered strain to improve antitumor effects. A) The loading effect of engineered strains. B) The polarization state of neutrophils after incubation with engineered strains for 1.5 h. C) 3 µm transwell inserts were used to detect the chemotactic ability of loaded engineered strains neutrophils. D,E) Killing activity of loaded engineered strains neutrophils in response to tumor cells measured by Annexin V/PI stain. F,G) The released VNP‐PD1nb from neutrophils could induce B16F10 apoptosis. H) The experiment scheme of B16F10 lung metastasis model treatment: 2 × 10^5^ B16F10 cells were injected via tail vein (Day 0); then 1 × 10^6^ NE(VNP) or 3 × 10^6^ VNP were injected via tail vein (Day 3); next, mice were sacrificed and organs were collected (Day 14). I) The picture of the lung after administration. J) The number of metastasis foci. K) The proportion of tumor‐infiltrating CD8^+^ T‐cells in CD45^+^ cells. L,M) Similarly, the proportion of CD69^+^CD8^+^ T‐cells in tumor was shown in (L) and (M). N) The percentage of cytotoxic CD8^+^ T‐cells (Granzyme B^+^) in tumor. Data are shown as the mean ± SD. **** *p* < 0.0001, *** *p* < 0.001, ** *p* < 0.01, * *p* < 0.05, ns: no significance.

After characterizing VNP‐PD1nb, the loading efficiency of VNP‐PD1nb was measured, and compared with that of VNP, a 1.5‐fold increase was observed (**Figure** **5**A). Similarly, the effect of VNP‐PD1nb on the stimulation of neutrophil polarization was evaluated by RT‐PCR (Figure 5B). The results indicated that the levels of the antitumor markers *ICAM‐1*, *CCL3*, *IL‐1β*, and *TNF‐α* were significantly increased (≈10–20 times); in contrast, the levels of protumor markers such as *CCL2*, *CCL5*, and *Arg‐1* were decreased. In addition, we examined the polarization of macrophages after incubation with VNP‐PD1nb to evaluate the immune‐active ability of VNP‐PD1nb (Figure S13A–E, Supporting Information). By the same token, VNP‐PD1nb stimulated the polarization of M1‐like macrophages. Next, the chemotactic ability of NE(PD1nb) was detected (Figure 5C). Concordant with the NE(VNP), the NE(PD1nb) had normal chemotactic ability. As illustrated in Figure 5D,E, NE(PD1nb) induced approximately twofold more B16F10 cell apoptosis than NE(VNP), which suggests that NE(PD1nb) may have a better tumor‐killing capacity in vivo. In addition, to confirm that intracellular VNP‐PD1nb (VNP‐PD1nb_R_) had tumor‐killing ability in vivo, VNP‐PD1nb_R_ was collected and incubated with B16F10 cells, and it was determined that VNP‐PD1nb_R_ had enhanced tumor‐killing ability (Figure 5G,F). In keeping with the observation above, VNP‐PD1nb_R_ had normal immune activity, which was highlighted after treating polarized type RAW264.7 macrophages with VNP‐PD1nb_R_ (Figure S13F,G, Supporting Information).

To explore the therapeutic efficacy of the engineered strain, NE(PD1nb) were used to treat the B16F10 lung metastasis model mice, the treatment strategy is shown in Figure 5H. As shown in Figure 5I,J, the number of metastatic foci in the NE(PD1nb) group was significantly decreased by approximately tenfold compared with that in the untreated PBS group. Notably, compared with VNP‐PD1nb, NE(PD1nb) treatment led to a decrease in metastatic foci by approximately fourfold. Pictures of the spleen and H&E‐stained organs indicated that the NE(PD1nb) group had negligible side effects; indeed, the side effects were significantly reduced compared with those produced by VNP‐PD1nb (Figure S14A–C, Supporting Information).

### 2.7 NE(PD1nb) Stimulated CD4+ T‐Cells Maturity and Cytotoxic CD8+ T‐Cells Activation by DCs Tumor Antigen Presentation

After ensuring the therapeutic effect of NE(PD1nb) in vivo, the mechanism was explored by FACS, and the strategy is shown in Figure S15A (Supporting Information). First, the TME is shown in Figure 5K and Figure S16A–H (Supporting Information). The percentage of tumor‐infiltrating CD8^+^ T‐cells in the NE(PD1nb) group was significantly increased by approximately twofold compared with those in the other groups (Figure 5K). As shown in Figure S16E (Supporting Information), compared with the PBS group, the number of DCs significantly increased twofold after treatment with NE(PD1nb) and was higher than that observed in the VNP group. This phenomenon was also observed in macrophages (Figure S16F, Supporting Information). Treatment with NE(PD1nb) increased the number of neutrophils compared with PBS administration but there were slightly fewer neutrophils than that found in the VNP group (Figure S16G, Supporting Information). In addition, the number of tumor‐infiltrating CD4^+^ T‐cells showed no significant variation (Figure S16H, Supporting Information). To observe the distribution of infiltrating T‐cells in lung tissue, lung tissue sections were stained with immunofluorescence (Figure S17A, Supporting Information). The result indicates that CD8^+^ T‐cells tended to focus on tumor core (DAPI‐dense), by contrast, CD4^+^ T‐cells tended to evenly infiltrate in normal tissue (DAPI‐sparse) and tumor regions in NE(PD1nb) group. By contrary, the CD8^+^ T‐cells had much less infiltration and CD4^+^ T‐cells evenly distributed in PBS and NEs group. In the VNP and NE(VNP) group, CD4^+^ T‐cells distribution remained constant and CD8^+^ T‐cells number was increased slightly. For the VNP‐PD1nb group, CD8^+^ T‐cells clustered in tumor core, however, the number of CD8^+^ T‐cells was less than NE(PD1nb) group.

**Figure 6:**
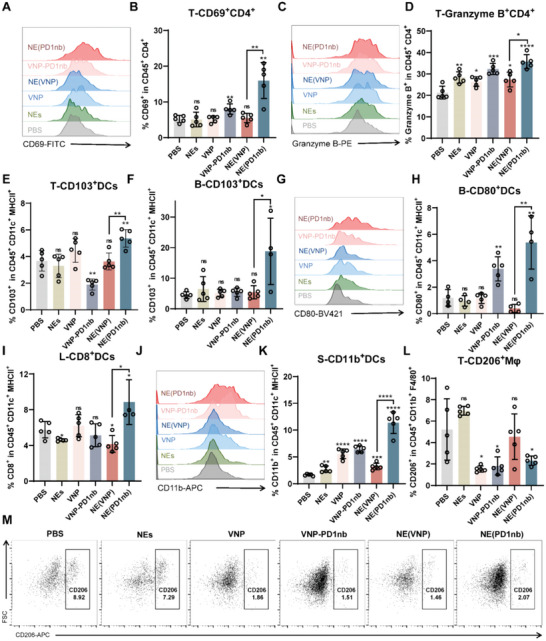
NE(PD1nb) stimulated T‐cells and macrophages activation and antitumor polarization. A,B) The expression level of CD69 in tumor‐infiltrating CD4^+^ T‐cells was detected by FACS. C,D) Similarly, the percentage of cytotoxic CD4 T‐cells (Granzyme B^+^) was evaluated, and the histogram plots were shown in (C), and the statistical diagrams were shown in (D). E) 5 days after NE(PD1nb) administration, the level of CD103 in DCs was determined in tumor. F) The percentage of CD103 positive cells in DCs in peripheral blood. G,H) Similarly, the expression level of CD80^+^ DCs in peripheral blood was measured by FACS. I) Within TdLN, the expression level of CD8^+^ in DCs. J) The FACS histogram plot of CD11b^+^DCs in the spleen. K) The statistic diagram of CD11b^+^DCs in the spleen. L,M) The percentage of “M2” macrophages (CD206^+^) in the tumor was measured. Data are shown as the mean ± SD. **** *p* < 0.0001, *** *p* < 0.001, ** *p* < 0.01, * *p* < 0.05, ns: no significance.

The expression level of CD69 in T cells is used to evaluate the maturation or activation of T cells. As shown in Figure 5L,M, NE(PD1nb) treatment induced more intertumoral CD69^+^ CD8^+^ T‐cells, threefold than that in the PBS group and twofold than that in the VNP‐PD1nb group. Except for the mature T cells, the proportion of cytotoxic T cells was evaluated after administration, which was represented by those highly expressing granzyme B. The proportion of granzyme B^+^ CD8^+^ T‐cells was significantly increased compared with that in the PBS group and the VNP‐PD1nb group by at least twofold (Figure 5N). In the NE(PD1nb) group, the tumor‐infiltrating CD4^+^ T‐cells showed higher expression of CD69 than those in the other groups by at least threefold (**Figure** **6**A,B). Similarly, enhanced expression of granzyme B in CD4^+^ T cell was found in the VNP‐PD1nb group and the NE(PD1nb) group and the percentage of granzyme B^+^ CD4^+^ T‐cells reached 32.34% and 35.7%, respectively (Figure 6C,D).

DCs are antigen‐presenting cells (APCs) that can activate the recruitment and stimulation of antitumor T cells. Based on this, we evaluated the state of DCs after treatment. In the NE(PD1nb) group, the polarized type of tumor‐infiltrating DCs was changed after administration, such as CD11b^+^ DCs (cDC2s), and CD8^+^ DCs (resident cDC1s),^[^ ^51^, ^52^ ^]^ and their levels significantly increased compared with those in the PBS group, which suggests that DCs were activated and regulated the tumor‐related immune response after treatment with NE(PD1nb) (Figure S18A,B, Supporting Information). The number of migratory cDC1s (CD103^+^ DCs) was significantly increased in tumors after NE(PD1nb) treatment (Figure 6E). As expected, the number of DCs was also significantly increased in peripheral blood (Figure S18C,D, Supporting Information). In addition, an increase in CD103^+^ DCs was observed in peripheral blood, approximately fourfold, compared with the PBS group and the VNP‐PD1nb group (Figure 6F). As discussed above, the numbers of CD4‐stimulated cDC2s and CD8‐stimulated cDC1s, including resident cDC1s and migratory cDC1s, were significantly increased in tumor and peripheral blood, which suggests that DCs were activated after NE(PD1nb) treatment. Based on this, the proportion of activated DCs was determined. As expected, the expression of CD80 in DCs was significantly upregulated by threefold compared with that in the PBS group and approximately two times higher than that in the VNP‐PD1nb group (Figure 6G,H).

In terms of the state of DCs in the spleen and the tumor‐draining lymph nodes (TdLNs), the proportion of mature DCs in the TdLNs in the NE(PD1nb) group was significantly increased by 5%, approximately five times higher than that in the PBS group (Figure S18E, Supporting Information). Concomitantly, NE(PD1nb) treatment induced an increase in CD8^+^ cDC1s in the TdLNs compared with those in the PBS group and VNP‐PD1nb group (Figure 6I). Notably, the proportion of CD11b^+^ cDC2s was the highest, with a fivefold increase compared with the PBS group and a twofold increase compared with the VNP‐PD1nb group (Figure 6J,K). Overall, NE(PD1nb) activated DC differentiation and maturation and stimulated tumor antigen presentation, thus exerting antitumor effects.

As the pool of T cells matures, the state of the T cells in the spleen and TdLNs is crucial for the antitumor response. As expected, CD69^+^ CD4^+^ T‐cells and CD69^+^ CD8^+^ T‐cells in the TdLN and spleen had an increasing trend, which was similar to the state of intertumoral T cells (Figures S18F,G and S19A,B, Supporting Information). Notably, the proportions of PD1^+^ CD8^+^ T‐cells and PD1^+^ CD4^+^ T‐cells in the spleen were significantly reduced by at least twofold compared with those in the PBS group, which suggests that protumoral T cells were reduced by stimulation with NE(PD1nb) (Figure S19C–F, Supporting Information).

### 2.8 NE(PD1nb) Stimulated the M1‐Like Differentiation of Macrophages

Macrophages as APCs and tumor‐killing cells play vital roles in the tumor immune response; however, tumors stimulate macrophages to differentiate into a protumor state.^[^ ^53^, ^54^ ^]^ In this scenario, changing the state of macrophages is a critical pathway in tumor therapy. As shown in Figure 6L,M, NE(PD1nb) treatment induced a significant reduction in the percentage of M2‐like macrophages, which were CD206‐positive. Notably, the number of M1‐like macrophages in the spleen was significantly increased in the NE(PD1nb) group, which was highlighted by the increase in the percentage of high‐expressing CD86 macrophages (Figure S20A,B, Supporting Information). In contrast, NE(PD1nb) treatment induced a decrease in CD206^+^ macrophages (at least twofold) compared with the PBS group, which suggests that the number of M2‐like macrophages in the spleen was significantly decreased after administration (Figure S20C, Supporting Information).

**Scheme 1:**
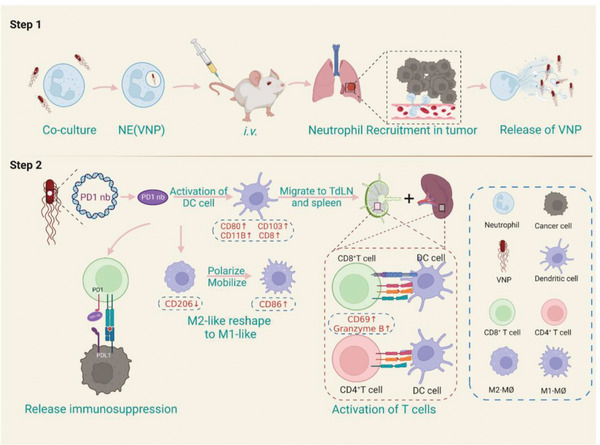
Schematic diagram of immunoregulation of B16F10‐bearing mice by NE(PD1nb). Step 1: VNP‐PD1nb were loaded by neutrophils in vitro; and NE(PD1nb) were injected intravenously and transported along the blood vessel; next, NE(PD1nb) colonized in tumor foci on the lung, and VNP‐PD1nb were released and proliferated in tumor. Step 2: PD1nb blocked PD1‐PDL1 axis and thus had a remission on immunosuppression. 1) NE(PD1nb) induced the reduction of M2‐like macrophages (CD206^+^) and polarized the increase of M1‐like macrophages (CD86^+^). 2) NE(PD1nb) stimulated DCs maturation manifesting as elevated CD80; the enhanced signals of DCs antigen presentation were upregulated, such as CD8, CD103, CD11b; 3) NE(PD1nb) activated CD8^+^ T‐cells and CD4^+^ T‐cells by antigen presentation which highlight by upregulating Granzyme B and CD69, and then stimulated cytotoxic T‐cell‐induced tumor apoptosis.

As previously shown, NE(PD1nb) treatment induced the remodeling of the immunosuppressive TME, including the differentiation of DCs, the activation of cytotoxic T cells, and the polarization of antitumor macrophages (**Scheme** **1**).

## 3 Discussion

Although VNP is the most promising oncolytic bacterium, the balance between its safety and therapeutic efficacy represents an urgent issue that needs to be properly addressed. To utilize this strategy, the tumor colonization efficiency of VNP needs to be enhanced. In this report, we demonstrate that neutrophils are a critical factor in tumor colonization by VNP. In line with our reports, early research revealed that depletion of host neutrophils leads to an increased titer of bacteria in the tumor.^[^ ^26^ ^]^ Owing to the phagocytic ability of neutrophils, neutrophils have been considered a vector for loading drugs and have been applied in the field of tumor therapy. Based on this, in our study, neutrophils acted as a vector for VNP, protecting VNP from the host immune system, homing VNP to the tumor core and then releasing live VNP. Moreover, neutrophils have the capability to colonize and reside in the lung; therefore, we designed a lung metastasis tumor‐targeting delivery system for VNP. In our delivery system, neutrophils could load 6.33 VNP per cell after 1.5 h of incubation, and VNP survival was maintained at a minimum of 30%. NE(VNP) could retain their normal activity and phagocytic and chemotaxis abilities. In addition, the release efficiency of VNP remained under 2% over 4 h, except under tumor‐induced conditions, which suggests that the loading system is stable. VNP can be released from neutrophils and proliferate normally. The in vivo experimental results revealed that NE(VNP) colonized to a greater extent in the tumor and produced better tumor therapeutic efficacy, as well as causing fewer side effects than VNP alone. Taken together, the results here suggest that this NE(VNP) controlled release delivery system addresses the balance between safety and therapeutic efficacy of VNP by using an autogenous vector. In addition, NE(VNP) significantly reduced splenic immune cell infiltration, which echoes the phenomenon of hepatosplenomegaly remission. This is due to the significant decrease in spleen‐infiltrating VNP, and most of the neutrophil‐loaded VNP localized to the tumor site. These results indicated that the NE(VNP) system increases tumor targeting and improves the therapeutic effects, maintaining the balance between therapeutic efficacy and safety. Notably, the NE(VNP) system could increase the tumor colonization of VNP in the lung metastasis area; therefore, compared with VNP, NE(VNP) reduced the number of lung metastatic foci. In view of these findings, NE(VNP) lifted the restriction of VNP in solid tumors and metastatic tumor therapy.

A previous study showed that *Salmonella* slows down infection by exploiting the PD1:PDL1 interaction to inhibit the CD8^+^ T‐cell response. In addition, macrophages and B cells upregulated PDL1 and PDL2 in the early infection phase, and in the late infection phase, the expression level of PD1 in CD8^+^ T‐cells was increased.^[^ ^55^ ^]^ With the development of immune checkpoint blockers (ICBs), PD1, as the most popular target, has been extensively applied in antitumor therapies. In our report, a PD1 nanobody was utilized in our system to synergistically enhance the therapeutic efficacy of NE(VNP). As expected, NE(PD1nb) activated DC differentiation, thus stimulating the capacity of tumor antigen presentation, which is consistent with previous reports.^[^ ^50^, ^56^, ^57^ ^]^ Similarly, NE(PD1nb) stimulated the M1‐like differentiation of macrophages and induced CD4^+^ T‐cell maturation and cytotoxic CD8^+^ T‐cell activation by DC tumor antigen presentation. Notably, the expression of PD1 in CD8^+^ T‐cells and CD4^+^ T‐cells was significantly downregulated after NE(PD1nb) administration, which validated our design concept.

Our findings reveal that VNP and neutrophils have a strong synergistic effect, which was highlighted by the increase in tumor‐killing efficiency and tumor proliferation inhibition by NE(VNP). In keeping with this observation, neutrophil‐based delivery of VNP will be welcomed and applied in our further study.

## 4 Experimental Section

### Mouse Model

Animal experiments followed the appropriate ethical guidelines of the Nanjing University Animal Care and Use Committee (IACUC‐2003167). Female C57BL/6J, 6–8 weeks, were purchased from Huachuang Sino Company (Nanjing, China) and housed under constant pathogen‐free conditions. To establish a melanoma lung metastases model, C57BL/6J mice were i.v. injected with 1 × 10^5^ B16F10 cells. After 3 days, 3 × 10^6^ CFU VNP, 1 × 10^6^ NEs, and 1 × 10^6^ NE(VNP) was injected respectably by tail vein into tumor‐bearing mice. Then 5 days after administration, the mice were executed and tumor, spleen, TdLNs, and peripheral blood were collected. The mice body weight was measured daily.

Whole peripheral blood samples were collected to detect aspartate aminotransferase (AST), alanine aminotransferase (ALT), creatinine (Scr), and blood urea nitrogen (BUN). These experiments were completed by Wuhan servicebio technology company (Wuhan, China).

### Cell Culture

B16F10 and B16F1 mouse melanoma cells, RAW 264.7 mouse macrophage cells, and MEFs were archived in the laboratory. B16F10 and B16F1 were cultured in RPMI‐1640 medium (BBI, China) containing 10% fetal bovine serum (FBS, HyClone, USA). RAW 264.7 cells and MEF were cultured in Dulbecco's modified Eagle medium (BBI, China) containing 15% FBS (Gibco, USA). All the cell lines were cultured at 37 °C in an incubator with 5% CO_2_.

### Bacteria Strains and Plasmid

VNP20009 (VNP), VNP20009 expressing PD1 nanobody with J23100 promoter initiation (VNP‐PD1nb), VNP20009 expressing RFP or EGFP with J23100 promoter initiation (VNP‐RFP/EGFP), and VNP20009 expressing LuxCDABE with J23100 promoter initiation (VNP‐Lvx) were cultured in LB broth or on LB agar plates, at 37 °C. The microplate reader was used to measure bacteria growth curve constantly. All plasmids were constructed by using the ClonExpress II/MultiS One Step Cloning Kit (C112/C113, Vazyme).

The morphogens of bacteria were detected by scanning electron microscopy.

When OD_600_ reached 0.6–0.8 (logarithmic phase), the expression of the protein in bacteria was measured by the sonication of lysing bacteria.

### Isolation of Peritoneal Neutrophil and Neutrophil Depletion

The mice used to extract neutrophils belonged to the same batch as the line selected in the pharmacodynamics experiment. 4–6 h before isolation of peritoneal neutrophil, mice were subcutaneously (s.c.) injected with 1 mL nutrient broth to stimulate peritoneal neutrophil maturity. Neutrophils were isolated using a Percoll gradient and identified by FACS, then plated at a density of 1 × 10^6^ cells mL^−1^.

For the depletion of neutrophil, anti‐Ly6G antibody (200 µg mL^−1^, BE0075, BioXCell) and anti‐mouse IgG2b (BE0086, BioXCell) were used as an isotype and were subcutaneously injected twice. 24 h after injection, the proportion of Ly6g^+^CD11b^+^CD45^+^ cells was measured in peripheral blood to confirm the efficiency of neutrophil depletion.

### Neutrophil Phagocytosing VNP and Fluorescent Microbeads

Neutrophils were stimulated with 5 × 10^−3^ m GSK484 (an inhibitor of peptidyl arginine deiminase 4 (PAD4), the key gene of neutrophil extracellular traps production) (HY‐100514, MCE) for 30 min. After that, VNP (MOI = 100:1) were incubated with neutrophil for 0.5, 1.5, or 2.5 h at 37 °C in an incubator with 5% CO_2_. And then, 45 µg mL^−1^ gentamicin sulfate (B540724‐0010, Sangon Biotech) was added to the medium for 1 h, owing to killing bacteria on neutrophil surfaces.

To measure the phagocytosis efficiency of neutrophil after loading VNP, fluorescent microspheres (L2778‐1ML; Sigma‐Aldrich) were incubated with neutrophil that had loaded VNP (NE(VNP)) (at 0.5, 1.5, or 2.5 h, MOI = 100:1) at 2 h. Next, the ratio of VNP‐EGFP and beads‐PE in neutrophil was detected by FACS.

### Neutrophil‐Released VNP Assays

NE(VNP) were cocultured with B16F1, B16F10, MEF, or not at 0.5, 1, 2, or 4 h, respectively, at 37 °C in an incubator with 5% CO_2_ and supernatants were collected to count the numbers of released VNP by plate on LB plates. To get control group bacteria numbers, neutrophils after loading VNP were lysed by 1% Triton X‐100 at 10 min and planted on LB agar after being diluted in PBS. The percentage of released bacteria was counted by the followed formula

(1) $$\%\;=\;\frac{\;\mathrm{The}\;\mathrm{bacterial}\;\mathrm{numbers}\;\mathrm{of}\;\mathrm{experimental}\;\mathrm{groups}}{\mathrm{The}\;\mathrm{bacterial}\;\mathrm{numbers}\;\mathrm{of}\;\mathrm{control}\;\mathrm{group}}\times 100\%$$

To measure released bacteria proliferative activity and stimulative ability, the released VNP were collected from neutrophil and the bacteria growth curves were detected or cocultured with B16F10 or RAW264.7 cells, which evaluated the apoptosis of B16F10 and RAW264.7 polarization response.

### ROS and NO Measurement

After neutrophil loaded VNP at 0.5, 1.5, or 2.5 h, ROS detection kit (S0033S, Beyotime) and DAF‐FM DA (NO fluorescent probe, S0019, Beyotime) were used to measure neutrophil ROS and NO and followed the manufacturer's recommendations.

### Neutrophil Chemotaxis Assay

Neutrophil chemotaxes were performed using a 3 µm pore size transwell (3415, Corning). The lower chambers were added fMLP (10 × 10^−9^ m, HY‐P0224, MCE) or pre‐planted with 1 × 10^6^ per well RAW cells, the upper chambers were plated with 5 × 10^5^ per well neutrophils or NE(VNP), and cocultured for 2 h. The supernatants of lower chambers were collected to count neutrophils numbers and chemotactic ratios.

### Cell Proliferation and Cell Apoptosis Detection

Tumor cells proliferation after treatments was measured by B16F10‐GFP assay. Briefly, B16F10 cells were seeded at a density of 1 × 10^5^ per well in 24‐well plates and measured per 12 h by microplate reader (488–510 nm) to count the proportions of B16F10 proliferation.

The apoptosis levels of B16F10 were determined using a kit developed in the lab. 1 µL Annexin V‐APC (1 mg mL) and 1 µL PI‐PE (1 mg mL^−1^) were incubated with 1 × 10^6^ cells in binding buffer (10 × 10^−3^ m HEPES, 140 × 10^−3^ m NaCl, 2.5 × 10^−3^ m CaCl_2_) for 30 min at 4 °C. The stained cells were analyzed using FACS (NovoCyte Flow Cytometer (ACEA@)). The results were analyzed using FlowJo VX software.

### RT‐PCR

Total RNA was isolated using TRIzol Reagent (Vazyme, China). The cDNA templates were reverse transcribed from 1 µg RNA with a TUYOBO cDNA Synthesis kit (TUYOBO, Japan) according to the manufacturer's instructions. Relative mRNA levels were detected using one step RT‐PCR SYBR Green Kit (Vazyme, China) following the manufacturer's instructions. The primers were synthesized by Genscript (Nanjing, China) and are listed in Supporting Information, Table S1.

### Western Blot

Bacteria were harvested until growth to the logarithmic phase and disrupted using sonication and the supernatants were collected. All protein samples were subjected to 12% sodium dodecyl sulfate‐polyacrylamide gel electrophoresis and transferred to polyvinylidene fluoride membranes. The western blot antibody used was as follows: ANTI‐FLAG M2 antibody (B3111‐1MG) from Sigma (USA).

### Flow Cytometry

5 days after treatments, tumor‐bearing mouse tissues were collected. Spleen and TdLN were homogenized with 1 mL PBS to obtain single‐cell suspensions. Peripheral blood lymphocytes were obtained from peripheral blood. Tumors in lung were shredded and then digested with mixed medium (1 mg mL^−1^ Collagenase I, 1 mg mL^−1^ Collagenase IV, 200 µg mL^−1^ DNase I) at 37 °C for 40 min. All tissues were lysed with red blood cell lysis buffer (Beyotime, Nanjing) and then the cell suspensions were passed through a 200‐mesh filter.

The single‐cell suspensions were incubated in 1% bovine serum albumin for 15 min at 4 °C and stained with the following antibodies for 30 min at 4 °C (detailed antibody information is provided in the Supporting Information, Table S2). The stained cells were analyzed using flow cytometer (BD@ FACS Canto II systems). The results were analyzed using FlowJo VX software.

### Immunofluorescence and H&E

NE(VNP) were incubated with 100 × 10^−3^ m 2‐azido‐2‐deoxy‐D‐glucose (A886786‐250 mg, Macklin) for 1 h and were marked by DBCO‐CY5 (777374‐1MG, Sigma‐Aldrich) for 30 min. Subsequently, B16F10‐bearing mice were injected i.v. with 3 × 10^7^ CFU VNP or 1 × 10^7^ NE(VNP), after 1 or 2 h the tumor tissues were collected to prepare tissue sections. Tissue sections were stained with DAPI and full sections scan and H&E were completed by Wuhan servicebio technology company (Wuhan, China).

### Tumor Targeting Assay

After administrations, tumor tissues and organs were collected and lysed using 1% Triton X‐100 at 1 h at 4 °C. The supernatants were planted on LB agar after diluting in PBS, and the bacteria numbers were calculated.

### ELISA

7 days after administrations, the blood serum of tumor‐free mice and lung metastasis mice were collected to detect IgG level, which followed the Mouse IgG ELISA Kit instructions (EK271‐96, MULTI SCIENCE, China).

### Statistical Analysis

Results were expressed as the mean ± SD as specified. Mean differences were compared using *t*‐test or one‐way analysis of variance. A value of *p* < 0.05 was regarded as statistically significant. Data were analyzed with GraphPad Prism 8.3 software. (**** *p* < 0.0001, *** *p* < 0.001, ** *p* < 0.01, * *p* < 0.05.)

## Conflict of Interest

The authors declare no conflict of interest.

## Supporting information

Supporting Information

## Data Availability

The data that support the findings of this study are available in the supplementary material of this article.

## References

[1] S. Zhou , C. Gravekamp , D. Bermudes , K. Liu , Nat. Rev. Cancer 2018, 18, 727. 30405213 10.1038/s41568-018-0070-z PMC6902869

[2] N. S. Forbes , R. S. Coffin , L. Deng , L. Evgin , S. Fiering , M. Giacalone , C. Gravekamp , J. L. Gulley , H. Gunn , R. M. Hoffman , B. Kaur , K. Liu , H. K. Lyerly , A. E. Marciscano , E. Moradian , S. Ruppel , D. A. Saltzman , P. J. Tattersall , S. Thorne , R. G. Vile , H. H. Zhang , S. Zhou , G. McFadden , J. Immunother. Cancer 2018, 6, 78. 30081947 10.1186/s40425-018-0381-3 PMC6091193

[3] Y. Chen , X. Liu , Y. Guo , J. Wang , D. Zhang , Y. Mei , J. Shi , W. Tan , J. H. Zheng , Acta Biomater. 2021, 124, 72. 33561563 10.1016/j.actbio.2021.02.006

[4] K. Liang , Q. Liu , P. Li , H. Luo , H. Wang , Q. Kong , Cancer Lett. 2019, 448, 168. 30753837 10.1016/j.canlet.2019.01.037

[5] S. Li , H. Yue , S. Wang , X. Li , X. Wang , P. Guo , G. Ma , W. Wei , Adv. Drug Delivery Rev. 2022, 188, 114444. 10.1016/j.addr.2022.114444 35817215

[6] Y. Guo , Y. Chen , X. Liu , J. J. Min , W. Tan , J. H. Zheng , Cancer Lett. 2020, 469, 102. 31666180 10.1016/j.canlet.2019.10.033

[7] Z. Mi , Z.‐C. Feng , C. Li , X. Yang , M.‐T. Ma , P.‐F. Rong , J. Cancer 2019, 10, 4765. 31598148 10.7150/jca.32650 PMC6775532

[8] K. C. L. C. Clairmont , J. Pike , M. Ittensohn , K. B. Low , J. Pawelek , D. Bermudes , S. M. Brecher , D. Margitich , J. Turnier , Z. Li , X. Luo , I. King , L. M. Zheng , J. Infect. Dis. 2000, 181, 1996. 10837181 10.1086/315497

[9] V. J. G. By John , F. Toso , P. Hwu , F. M. Marincola , N. P. Restifo , D. J. Schwartzentruber , S. L. T. Richard , M. Sherry , J. C. Yang , F. Stock , L. J. Freezer , K. E. Morton , C. Seipp , S. M. Leah Haworth , D. White , S. MacDonald , J. Mao , M. Sznol , A. Rosenberg , J. Clin. Oncol. 2002, 20, 142. 11773163 10.1200/JCO.2002.20.1.142 PMC2064865

[10] C. Zhao , J. He , H. Cheng , Z. Zhu , H. Xu , J. Exp. Clin. Cancer Res. 2016, 35, 107. 27371094 10.1186/s13046-016-0381-4 PMC4930618

[11] X. Zhang , X. Cheng , Z.‐C. Hua , Oncotarget 2016, 7, 12. 10.18632/oncotarget.7496 PMC4924763 26910836

[12] B. F. Sieow , K. S. Wun , W. P. Yong , I. Y. Hwang , M. W. Chang , Trends Cancer 2021, 7, 447. 33303401 10.1016/j.trecan.2020.11.004

[13] W. Yoon , Y. Yoo , Y. S. Chae , S. H. Kee , B. M. Kim , Ann. Oncol. 2018, 29, 2010. 30016386 10.1093/annonc/mdy240

[14] W. Yoon , Y. C. Park , J. Kim , Y. S. Chae , J. H. Byeon , S. H. Min , S. Park , Y. Yoo , Y. K. Park , B. M. Kim , Eur. J. Cancer 2017, 70, 48. 27883926 10.1016/j.ejca.2016.10.010

[15] Y. Chen , M. Du , Z. Yuan , Z. Chen , F. Yan , Nat. Commun. 2022, 13, 4468. 35918309 10.1038/s41467-022-31932-x PMC9345953

[16] W. Chen , C. He , N. Qiao , Z. Guo , S. Hu , Y. Song , H. Wang , Z. Zhang , B. Ke , X. Sun , Biomaterials 2022, 286, 121582. 35609407 10.1016/j.biomaterials.2022.121582

[17] W. Wang , H. Xu , Q. Ye , F. Tao , I. Wheeldon , A. Yuan , Y. Hu , J. Wu , Nat. Biomed. Eng. 2022, 6, 44. 35058589 10.1038/s41551-021-00834-6

[18] F. Chen , Z. Zang , Z. Chen , L. Cui , Z. Chang , A. Ma , T. Yin , R. Liang , Y. Han , Z. Wu , M. Zheng , C. Liu , L. Cai , Biomaterials 2019, 214, 119226. 31174068 10.1016/j.biomaterials.2019.119226

[19] M. Wu , W. Wu , Y. Duan , X. Li , G. Qi , B. Liu , Chem. Mater. 2019, 31, 7212.

[20] W. Chen , Y. Wang , M. Qin , X. Zhang , Z. Zhang , X. Sun , Z. Gu , ACS Nano 2018, 12, 5995. 29786420 10.1021/acsnano.8b02235

[21] J. X. Fan , M. Y. Peng , H. Wang , H. R. Zheng , Z. L. Liu , C. X. Li , X. N. Wang , X. H. Liu , S. X. Cheng , X. Z. Zhang , Adv. Mater. 2019, 31, 1808278. 10.1002/adma.201808278 30803049

[22] T. Harimoto , J. Hahn , Y. Y. Chen , J. Im , J. Zhang , N. Hou , F. Li , C. Coker , K. Gray , N. Harr , S. Chowdhury , K. Pu , C. Nimura , N. Arpaia , K. W. Leong , T. Danino , Nat. Biotechnol. 2022, 40, 1259. 35301496 10.1038/s41587-022-01244-y PMC9371971

[23] Z. Mi , L. Guo , P. Liu , Y. Qi , Z. Feng , J. Liu , Z. He , X. Yang , S. Jiang , J. Wu , J. Ding , W. Zhou , P. Rong , Nano Lett. 2021, 21, 414. 33356313 10.1021/acs.nanolett.0c03811

[24] M. Zhang , N. S. Forbes , J. Controlled Release 2015, 199, 180. 10.1016/j.jconrel.2014.12.014 PMC4308568 25523033

[25] S. Ganai , R. B. Arenas , J. P. Sauer , B. Bentley , N. S. Forbes , Cancer Gene Ther. 2011, 18, 457. 21436868 10.1038/cgt.2011.10 PMC3117926

[26] K. Westphal , S. Leschner , J. Jablonska , H. Loessner , S. Weiss , Cancer Res. 2008, 68, 2952. 18413765 10.1158/0008-5472.CAN-07-2984

[27] J. Chen , Y. Qiao , B. Tang , G. Chen , X. Liu , B. Yang , J. Wei , X. Zhang , X. Cheng , P. Du , W. Jiang , Q. Hu , Z. C. Hua , Theranostics 2017, 7, 2250. 28740548 10.7150/thno.18816 PMC5505057

[28] P. X. Liew , P. Kubes , Physiol. Rev. 2019, 99, 1223. 30758246 10.1152/physrev.00012.2018

[29] D. Chu , X. Dong , X. Shi , C. Zhang , Z. Wang , Adv. Mater. 2018, 30, 1706245. 10.1002/adma.201706245 PMC6161715 29577477

[30] J. Che , A. Najer , A. K. Blakney , P. F. McKay , M. Bellahcene , C. W. Winter , A. Sintou , J. Tang , T. J. Keane , M. D. Schneider , R. J. Shattock , S. Sattler , M. M. Stevens , Adv. Mater. 2020, 32, 2003598. 10.1002/adma.202003598 PMC7613371 33103807

[31] J. Xue , Z. Zhao , L. Zhang , L. Xue , S. Shen , Y. Wen , Z. Wei , L. Wang , L. Kong , H. Sun , Q. Ping , R. Mo , C. Zhang , Nat. Nanotechnol. 2017, 12, 692. 28650441 10.1038/nnano.2017.54

[32] Y. Chen , K. Li , M. Jiao , Y. Huang , Z. Zhang , L. Xue , C. Ju , C. Zhang , Acta Pharm. Sin. B 2023, 13, 787. 36873164 10.1016/j.apsb.2022.08.012 PMC9978920

[33] Y. Wu , X. Han , R. Zheng , H. Cheng , J. Yan , X. Wu , Y. Hu , B. Li , Z. Wang , X. Li , H. Zhang , Nanoscale 2021, 13, 14825. 34533171 10.1039/d1nr04002b

[34] S. Li , M. Li , S. Huo , Q. Wang , J. Chen , S. Ding , Z. Zeng , W. Zhou , Y. Wang , J. Wang , Adv. Mater. 2021, 33, 2006160. 10.1002/adma.202006160 33296121

[35] Y. Xu , X. Zhang , G. Hu , X. Wu , Y. Nie , H. Wu , D. Kong , X. Ning , Biomaterials 2021, 279, 121224. 34710792 10.1016/j.biomaterials.2021.121224

[36] X. Yu , G. Xing , S. Sheng , L. Jin , Y. Zhang , D. Zhu , L. Mei , X. Dong , F. Lv , Adv. Sci. 2023, 10, e2207456. 10.1002/advs.202207456 PMC10214253 36967574

[37] D. F. Neil , S. Forbes , L. L. Munn , K. Jain , Cancer Res. 2003, 63, 5188. 14500342

[38] H. D. Lewis , J. Liddle , J. E. Coote , S. J. Atkinson , M. D. Barker , B. D. Bax , K. L. Bicker , R. P. Bingham , M. Campbell , Y. H. Chen , C. W. Chung , P. D. Craggs , R. P. Davis , D. Eberhard , G. Joberty , K. E. Lind , K. Locke , C. Maller , K. Martinod , C. Patten , O. Polyakova , C. E. Rise , M. Rudiger , R. J. Sheppard , D. J. Slade , P. Thomas , J. Thorpe , G. Yao , G. Drewes , D. D. Wagner , et al., Nat. Chem. Biol. 2015, 11, 189. 25622091 10.1038/nchembio.1735 PMC4397581

[39] J. S. Knight , V. Subramanian , A. A. O'Dell , S. Yalavarthi , W. Zhao , C. K. Smith , J. B. Hodgin , P. R. Thompson , M. J. Kaplan , Ann. Rheum. Dis. 2015, 74, 2199. 25104775 10.1136/annrheumdis-2014-205365 PMC4320672

[40] J. T. O. Cabrera , A. Makino , Pharmacol. Ther. 2022, 229, 107919. 34171333 10.1016/j.pharmthera.2021.107919 PMC8695637

[41] J. M. Adrover , J. A. Nicolas‐Avila , A. Hidalgo , Trends Immunol. 2016, 37, 334. 27083489 10.1016/j.it.2016.03.005

[42] A. C. Mihaila , L. Ciortan , R. D. Macarie , M. Vadana , S. Cecoltan , M. B. Preda , A. Hudita , A. M. Gan , G. Jakobsson , M. M. Tucureanu , E. Barbu , S. Balanescu , M. Simionescu , A. Schiopu , E. Butoi , Front. Immunol. 2021, 12, 708770. 34447377 10.3389/fimmu.2021.708770 PMC8384118

[43] A. Tyagi , S. Sharma , K. Wu , S. Y. Wu , F. Xing , Y. Liu , D. Zhao , R. P. Deshpande , R. B. D'Agostino Jr., K. Watabe , Nat. Commun. 2021, 12, 474. 33473115 10.1038/s41467-020-20733-9 PMC7817836

[44] M. E. Shaul , L. Levy , J. Sun , I. Mishalian , S. Singhal , V. Kapoor , W. Horng , G. Fridlender , S. M. Albelda , Z. G. Fridlender , Oncoimmunology 2016, 5, e1232221. 27999744 10.1080/2162402X.2016.1232221 PMC5139653

[45] Z. G. Fridlender , J. Sun , S. Kim , V. Kapoor , G. Cheng , L. Ling , G. S. Worthen , S. M. Albelda , Cancer Cell 2009, 16, 183. 19732719 10.1016/j.ccr.2009.06.017 PMC2754404

[46] Y. Chu , Y. Luo , B. Su , C. Li , Q. Guo , Y. Zhang , P. Liu , H. Chen , Z. Zhao , Z. Zhou , Y. Wang , C. Jiang , T. Sun , Acta Pharm. Sin. B 2023, 13, 298. 36815033 10.1016/j.apsb.2022.05.027 PMC9939302

[47] Z. Luo , Y. Lu , Y. Shi , M. Jiang , X. Shan , X. Li , J. Zhang , B. Qin , X. Liu , X. Guo , J. Huang , Y. Liu , S. Wang , Q. Li , L. Luo , J. You , Nat. Nanotechnol. 2023, 18, 647. 37081080 10.1038/s41565-023-01374-7

[48] A. J. Ozga , M. T. Chow , A. D. Luster , Immunity 2021, 54, 859. 33838745 10.1016/j.immuni.2021.01.012 PMC8434759

[49] N. Nagarsheth , M. S. Wicha , W. Zou , Nat. Rev. Immunol. 2017, 17, 559. 28555670 10.1038/nri.2017.49 PMC5731833

[50] C. Johansson , M. Ingman , M. J. Wick , Microb. Pathog 2006, 41, 49. 16782300 10.1016/j.micpath.2006.03.004

[51] S. K. Wculek , F. J. Cueto , A. M. Mujal , I. Melero , M. F. Krummel , D. Sancho , Nat. Rev. Immunol. 2020, 20, 7. 31467405 10.1038/s41577-019-0210-z

[52] S. Jhunjhunwala , C. Hammer , L. Delamarre , Nat. Rev. Cancer 2021, 21, 298. 33750922 10.1038/s41568-021-00339-z

[53] M. Sylvestre , C. A. Crane , S. H. Pun , Adv. Mater. 2020, 32, 1902007. 10.1002/adma.201902007 PMC7098849 31559665

[54] D. G. DeNardo , B. Ruffell , Nat. Rev. Immunol. 2019, 19, 369. 30718830 10.1038/s41577-019-0127-6 PMC7339861

[55] M. Lopez‐Medina , I. Carrillo‐Martin , J. Leyva‐Rangel , C. Alpuche‐Aranda , V. Ortiz‐Navarrete , Immunobiology 2015, 220, 1369. 26210046 10.1016/j.imbio.2015.07.005

[56] M. A. Tam , A. Rydström , M. Sundquist , M. J. Wick , Immunol. Rev. 2008, 225, 140. 18837781 10.1111/j.1600-065X.2008.00679.x

[57] F. Luo , X. Sun , Z. Qu , X. Zhang , World J. Microbiol. Biotechnol. 2016, 32, 22. 26745982 10.1007/s11274-015-1978-z

