# Supplementary material for: Neutrophil‐Mediated Tumor‐Targeting Delivery System of Oncolytic Bacteria Combined with ICB for Melanoma Lung Metastasis Therapy
Source: Adv Sci (Weinh). 2023 Aug 10;10(29):2301835. doi: 10.1002/advs.202301835 (PMC10582430; doi:10.1002/advs.202301835)
Supplement: Supplementary file 1 — Supporting Information [file ADVS-10-2301835-s001.pdf]

ADVANCED SCIENCE
Open Access

Supporting Information

for *Adv. Sci.*, DOI 10.1002/advs.202301835

Neutrophil-Mediated Tumor-Targeting Delivery System of Oncolytic Bacteria Combined with ICB for Melanoma Lung Metastasis Therapy

*Lina Liu, Wenjie Xin, Qiang Li, Baolian Huang, Te Yin, Siqi Hua, Chen Yang, Chen Chen, Chao Han and Zichun Hua**

Supporting Information

for *Adv. Sci.*

Neutrophil-mediated tumor-targeting delivery system of oncolytic bacteria combined with ICB for melanoma lung metastasis therapy

Lina Liu, Wenjie Xin, Qiang Li, Baolian Huang, Te Yin, Siqi Hua, Chen Yang, Chen Chen, Chao Han, Zichun Hua *

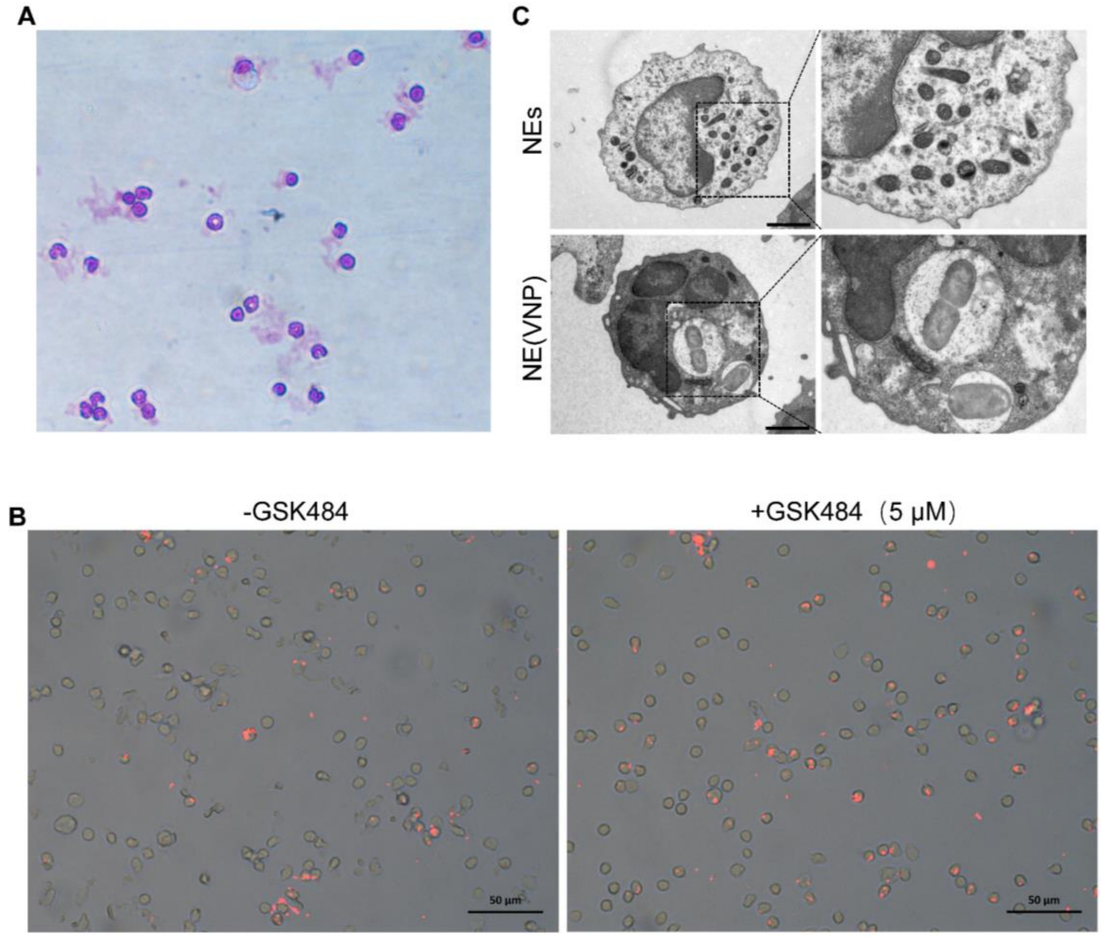

Figure S1. The purity of peritoneal neutrophil and the effect of GSK484. A) Giemsa stain was used to identify peritoneal neutrophils. B) The effect of GSK484 on neutrophil for 30 minutes. Scale bars: 50 μm. C) The TEM image of NE(VNP). Scale bars: 2 μm.

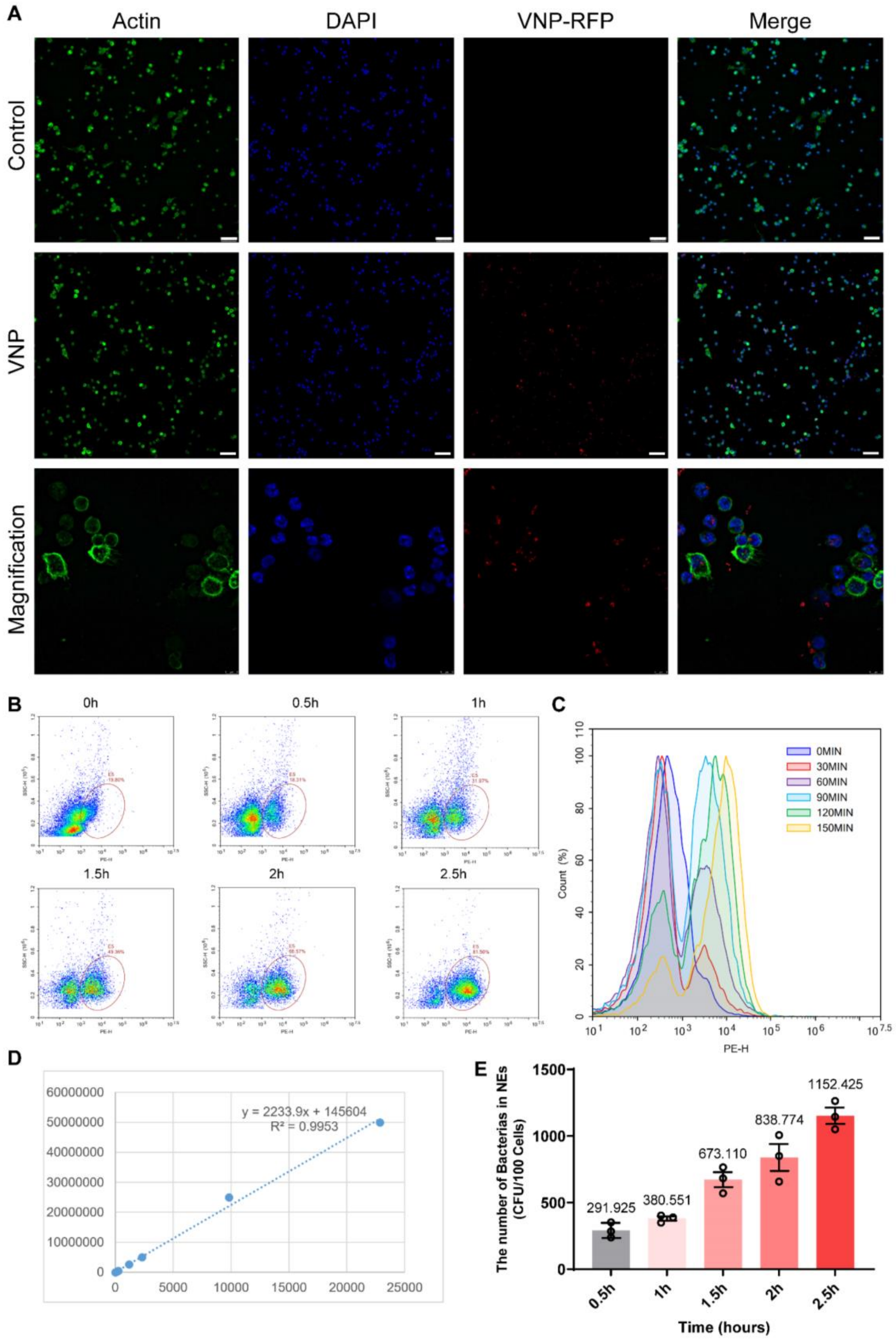

Figure S2. The efficiency of neutrophil loading VNP. A) The fluorescent pictures of NE(VNP); actin (green), DAPI (blue), VNP-RFP (red). Scale bars: 50 μm. B, C) The loading efficiency of neutrophil to VNP-RFP for different time points measured by

FACS. D) Regression line indicates the relationship of the MFI of VNP and VNP numbers. E) After incubated different times, the numbers of VNP per 100 cells.

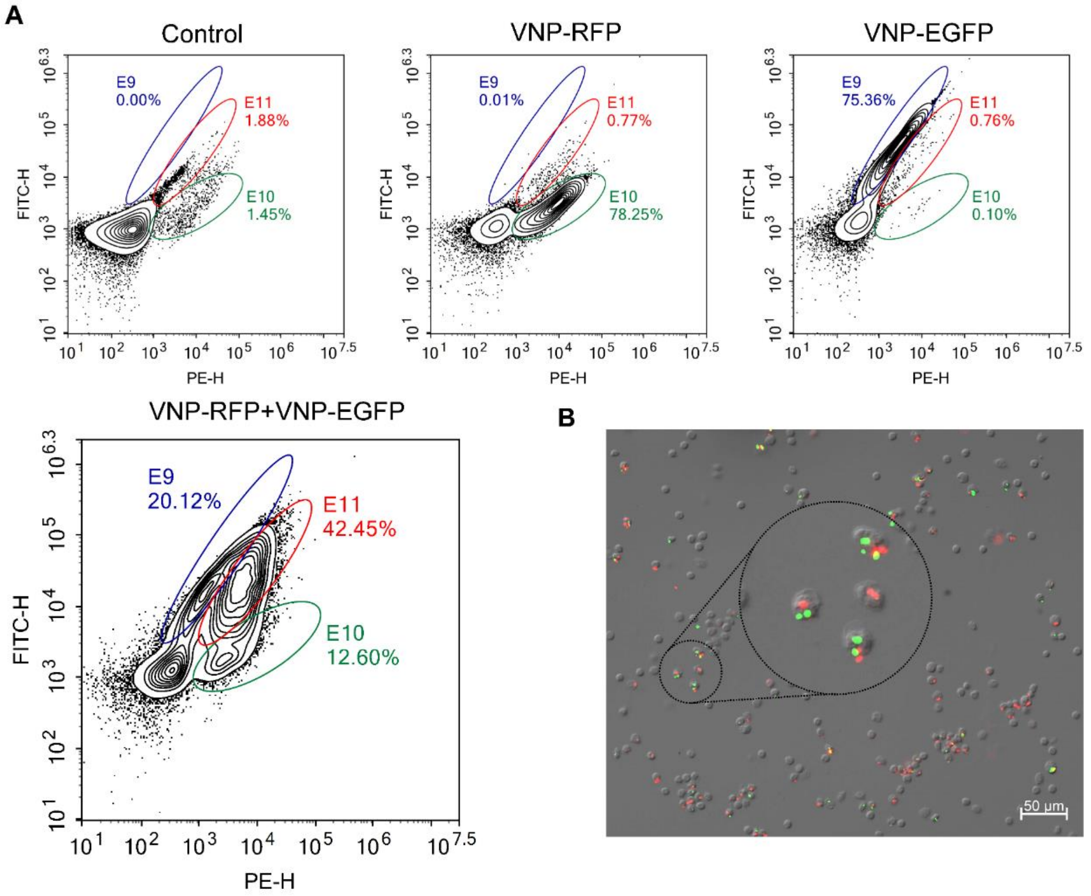

Figure S3. The efficiency of neutrophil simultaneous loading VNP-RFP and VNP-EGFP. A) The loading efficiency measured by FACS. B) The fluorescent picture of NE(VNP-RFP&VNP-EGFP).

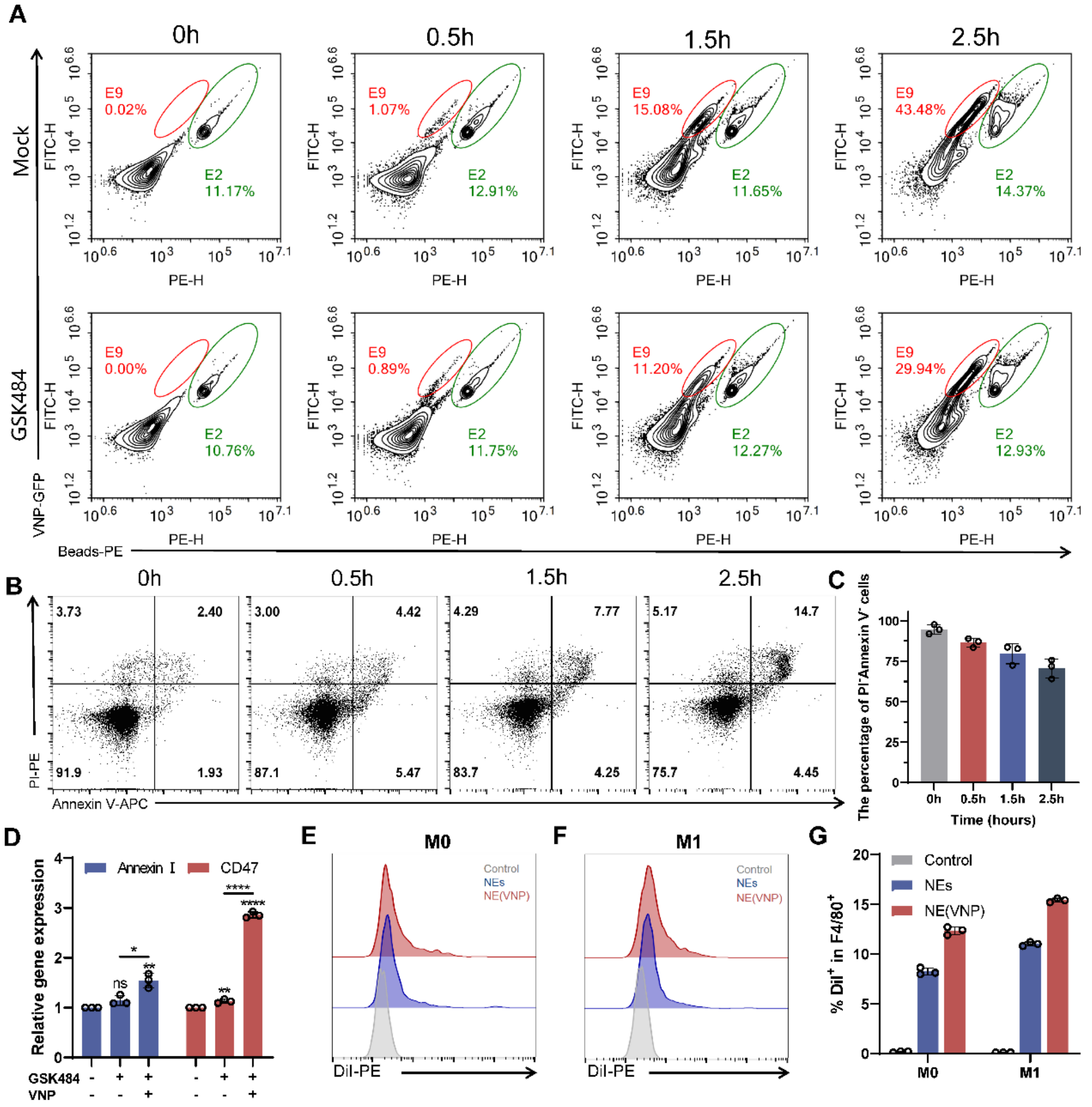

Figure S4. A) The phagocytic efficiency of NE(VNP) measured by FACS. B, C) The percentage of PS negative neutrophil was measured by Annexin V/PI staining after VNP loaded. D) The mRNA expression level of "eat-me" signal (*Annexin I*) and "do not eat-me" signal (*CD47*) in neutrophils after co-cultured with B16F10. E-G) The phagocytic efficiency of RAW264.7 (M0 and M1 type) 2 hours after incubation with DiI-labeled neutrophils. Data are shown as the mean ± SD. **** $p < 0.0001$, *** $p < 0.001$, ** $p < 0.01$, * $p < 0.05$.

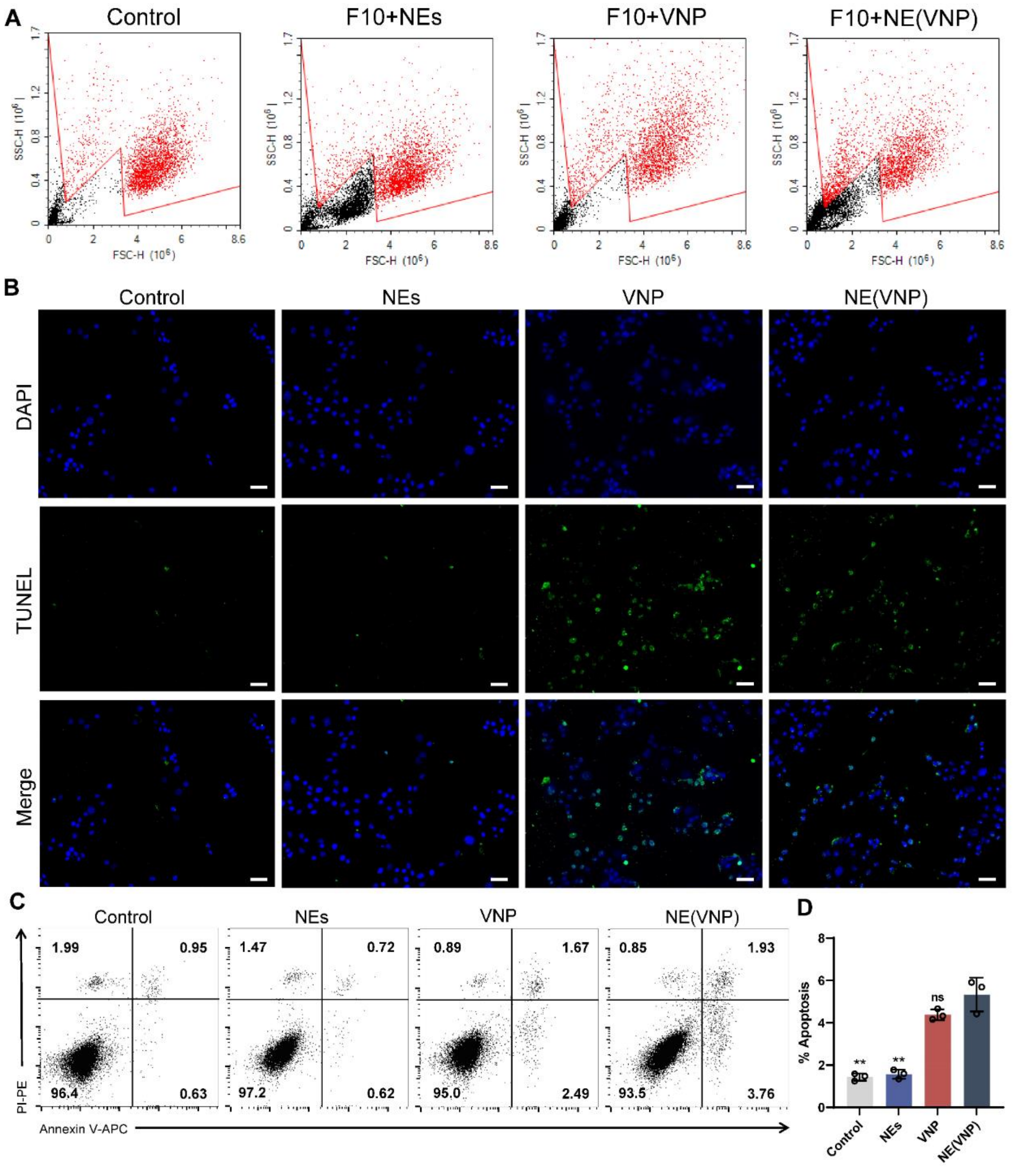

Figure S5. The killing activity of NE(VNP) to tumor cells. A) The FSC-SSC density plots gating strategy of NEs co-cultured with B16F10 cells in apoptosis assay experiments. B) The apoptosis levels of B16F10 after co-cultured with NE(VNP) determined by TUNEL stain. Scale bars: 25 μm. C, D) The apoptosis levels of B16F10 after incubated with the supernatant of cultured with NE(VNP) for 2 hours. Data are shown as the mean ± SD. **** $p < 0.0001$, *** $p < 0.001$, ** $p < 0.01$, * $p < 0.05$.

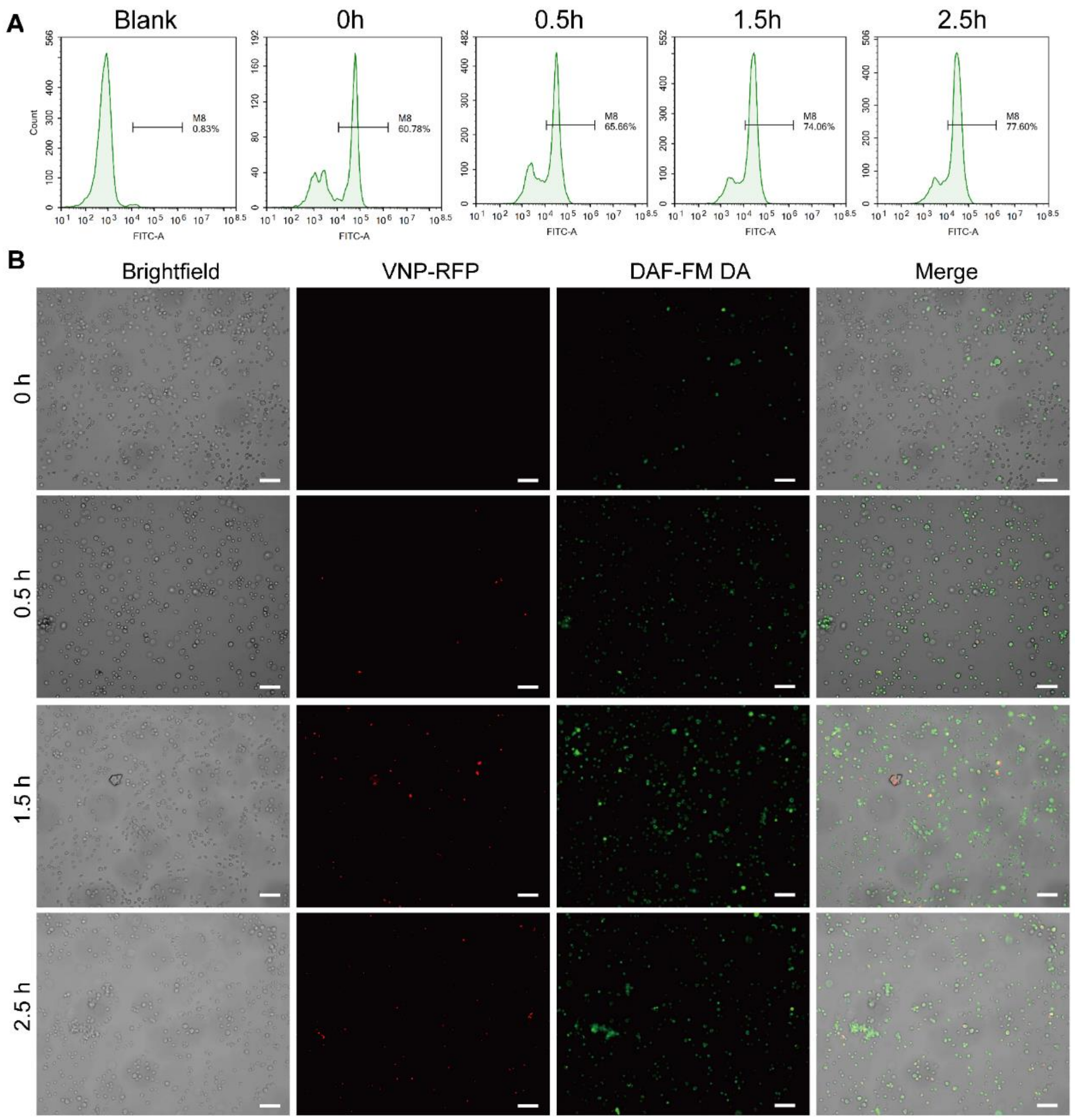

Figure S6. The NO levels of NE(VNP). A) The NO levels of NE(VNP) measured by FACS. B) The NO fluorescent picture of NE(VNP). Scale bars: 50 μm.

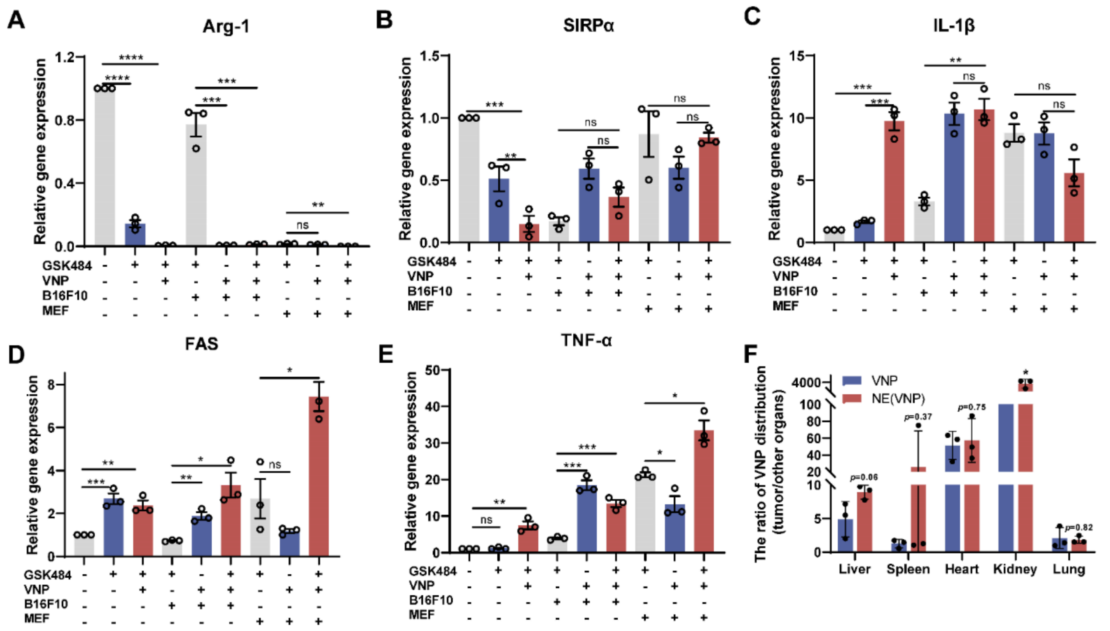

Figure S7. The polarity state of NE(VNP). A-C) The gene expression of anti-tumor gene, including to *FAS*, *TNF-α*, and *IL-1β*. D, E) The gene expression of anti-tumor gene, including to *Arg-1* and *SIRPα*. F) The ratio of tumor burden between other organs 72 hours after administrations. Data are shown as the mean ± SD. **** $p < 0.0001$, *** $p < 0.001$, ** $p < 0.01$, * $p < 0.05$.

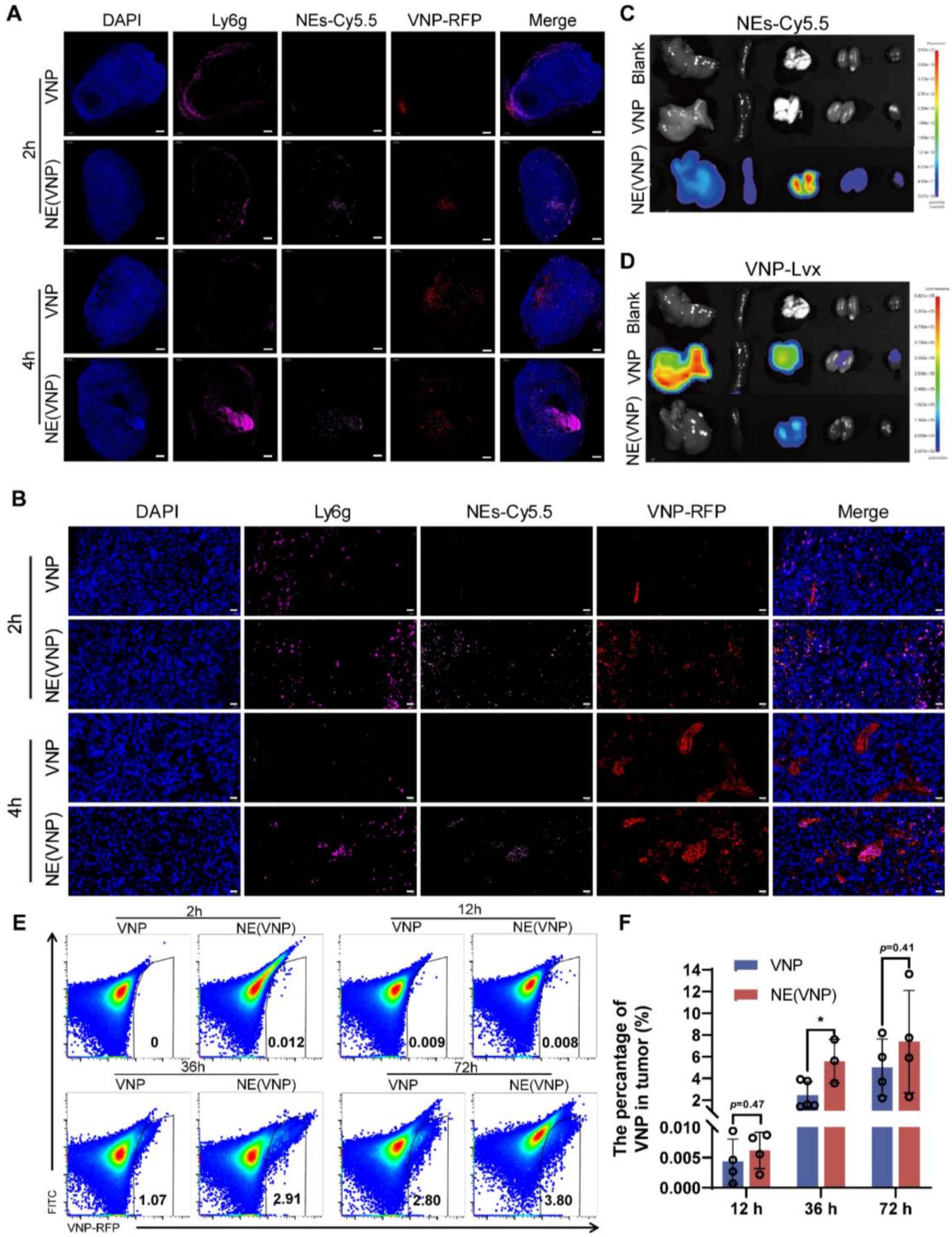

Figure S8. A) 3 × 10^7 CFU VNP-RFP and 1 × 10^7 NE(VNP)-Cy5.5 per mouse were *i.v.* injected into B16F10-bearing mouse, and tumor slices were prepared and scanned after 2 hours or 4 hours; DAPI (blue), Ly6g$^+$ cells (purple), NE-Cy5,5 (pink), VNP-RFP (red). Scale bars: 1000 μm. B) The magnified pictures of NE(VNP) targeting tumor core (40×); DAPI (blue), Ly6g$^+$ cells (purple), NE-Cy5,5 (pink), VNP-RFP (red), white arrowheads indicate Cy5,5$^+$ Ly6g$^+$ cells. Scale bars: 20 μm. C,

D) The animal imaging of mouse, which were injected Cy5.5 labeled NE(VNP-Lvx) and VNP-lvx for 2 hours. E, F) The percentage of VNP in the tumor after administrations at the different time points (2 hours, 12 hours, 36 hours, and 3 days) measured by FACS. The flow cytometry plots (E) and statistic diagram (F) of tumor infiltrating VNP. Data are shown as the mean ± SD. **** $p < 0.0001$, *** $p < 0.001$, ** $p < 0.01$, * $p < 0.05$.

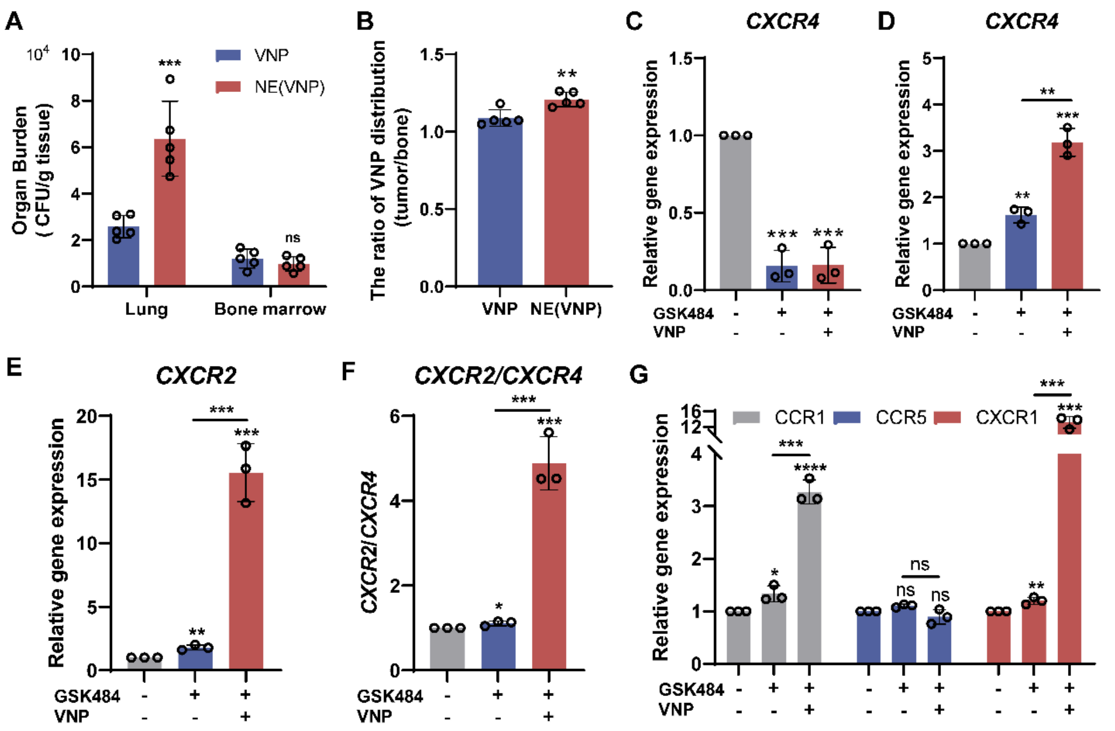

Figure. S9. The bone marrow chemotactic ability of NE(VNP). A) 12 hours after administrations, the titers of VNP in lung and bone marrow in the B16F10 lung metastasis model. B) The ratio of VNP organ burden in lung and bone marrow. C) The mRNA expression level of *CXCR4* in NE(VNP) and NEs. D, E) The mRNA expression level of *CXCR4* and *CXCR2* in NE(VNP) and NEs, which co-cultured with B16F10 at 4 hours. F) The ratio of expression level in *CXCR2* and *CXCR4*. G) After incubation with B16F10, the mRNA expression level of chemokine receptors (*CCR1*, *CCR5*, *CXCR1*) in neutrophils. Data are shown as the mean ± SD. **** $p < 0.0001$, *** $p < 0.001$, ** $p < 0.01$, * $p < 0.05$.

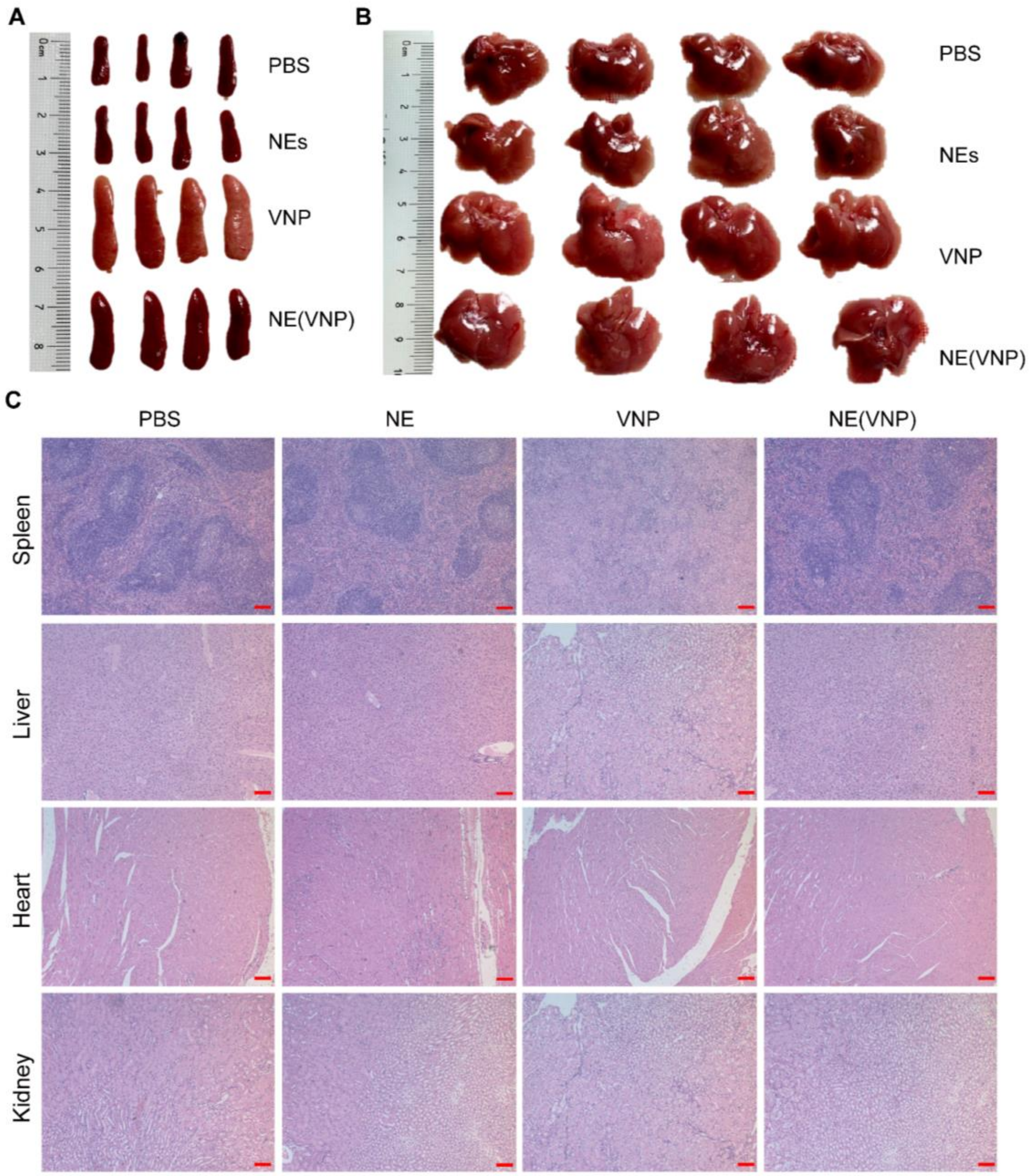

Figure S10. Biosafe of NE(VNP). A, B) The spleen and liver pictures of tumor-bearing mouse after 3 days administration. C) The organs HE stain of tumor-bearing mouse after 3 days administration. Scale bars: 200 μm.

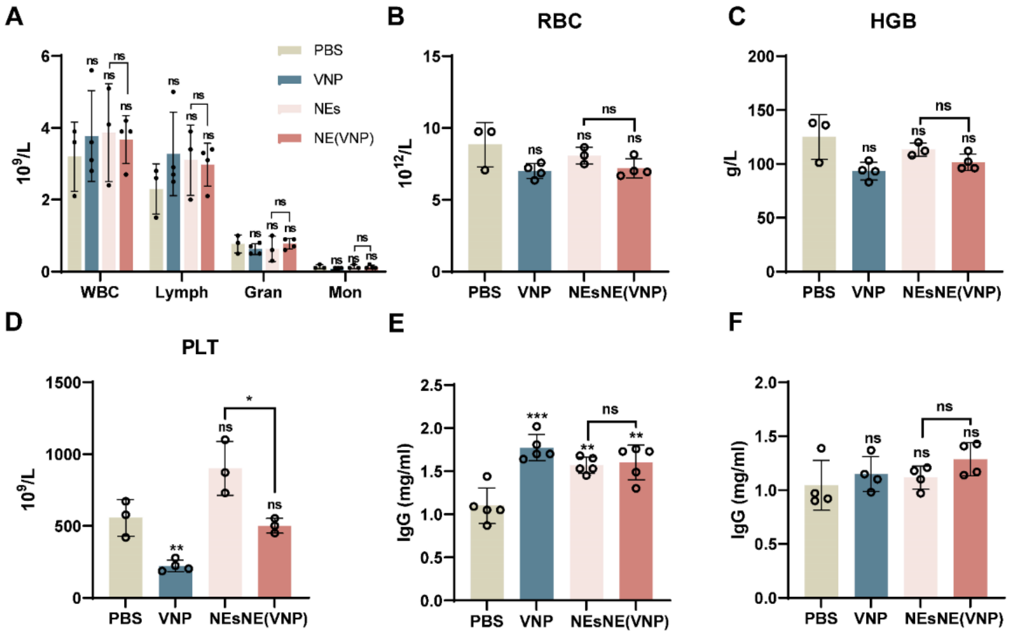

Figure. S11. The immunogenicity of NE(VNP). A-D) the results of blood routine 5 days after administrations. (n=3) E) Total immunoglobulin G (IgG) level 7 days after administrations in lung metastasis model. (n=5) F) Immunoglobulin G (IgG) level 7 days after administrations in normal mice. (n=4) Data are shown as the mean ± SD. **** $p < 0.0001$, *** $p < 0.001$, ** $p < 0.01$, * $p < 0.05$.

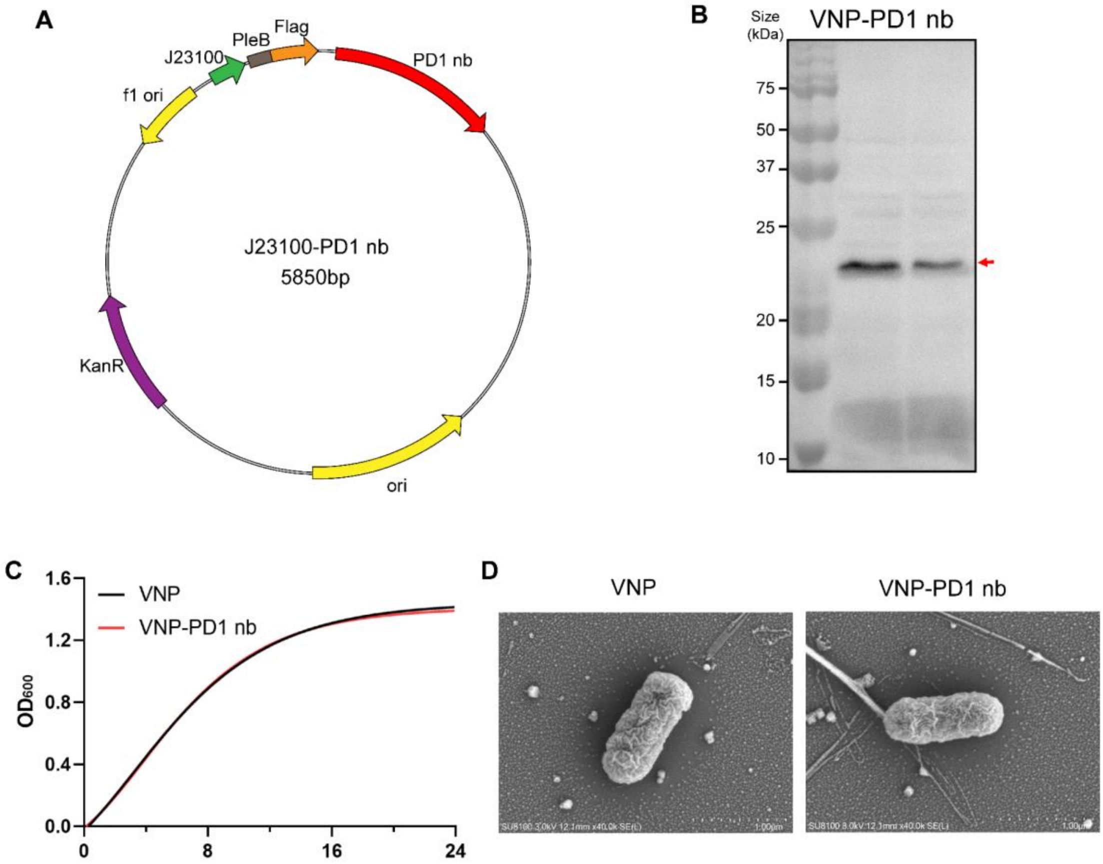

Figure S12. The phenotype of engineered strain. A) The expressed plasmid map of PD1nb. B) The expression of VNP-PD1nb. C) The growth curve of three engineered strains. D) The morphogen of engineered strain measured by SEM.

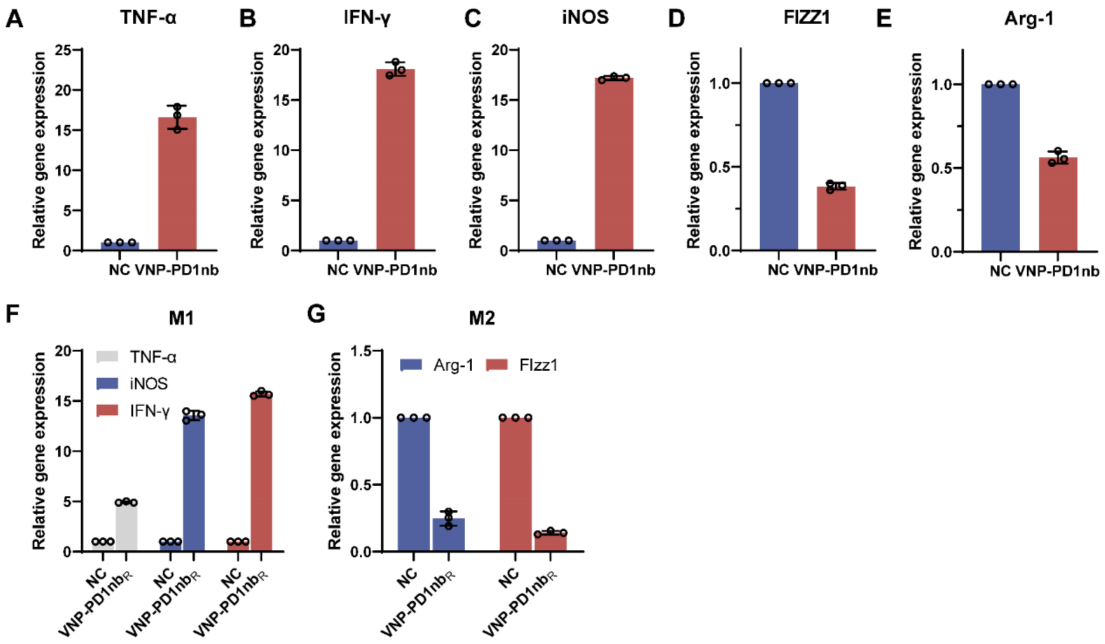

Figure S13. NE(PD1nb) induced macrophage polarization. A-E) 6 hours after VNP-PD1nb incubated with RAW264.7, RAW264.7 were collected to detect polarization by Real-time PCR, "M1" markers (*TNF-α, IFN-γ,* and *iNOS*) were showed in (A-C), and "M2" markers (*FIZZ1, Arg-1*) were showed in (D, E). F, G) Released VNP-PD1nb from neutrophil could kept ability to stimulate macrophage polarization.

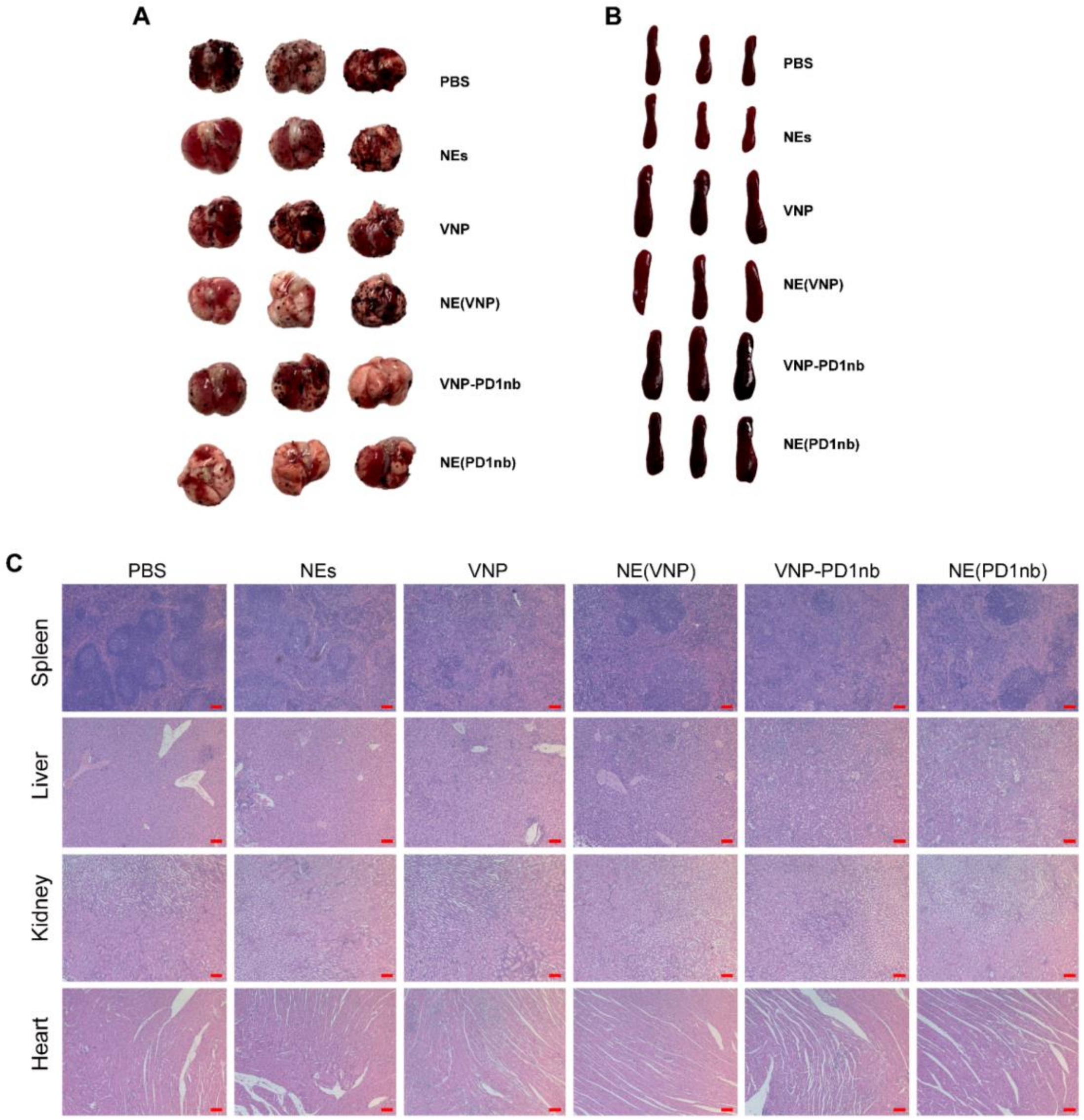

Figure S14. The efficacy and safety of NE(PD1nb). A) The picture of lung after treatments. B) The picture of spleen after treatments. C) H&E staining of tumor-bearing mouse organs. Scale bars: 200 μm.

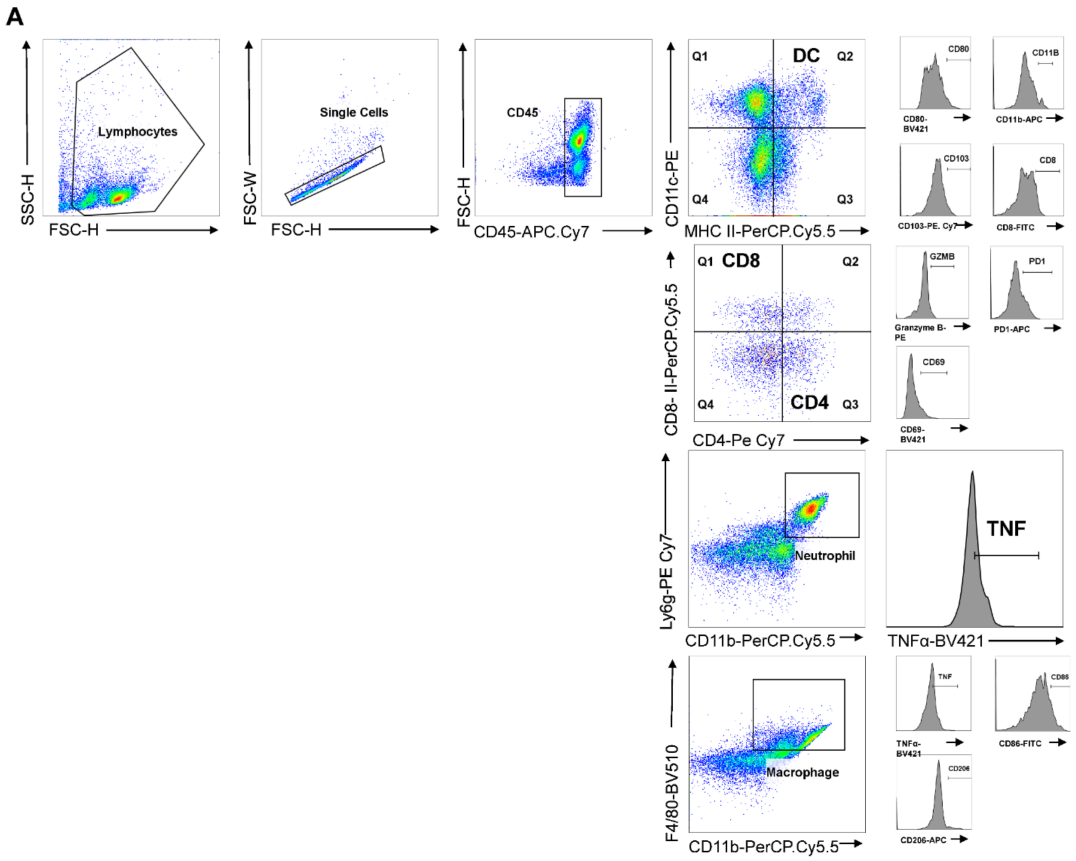

Figure S15. The plan of flow cytometry experiments.

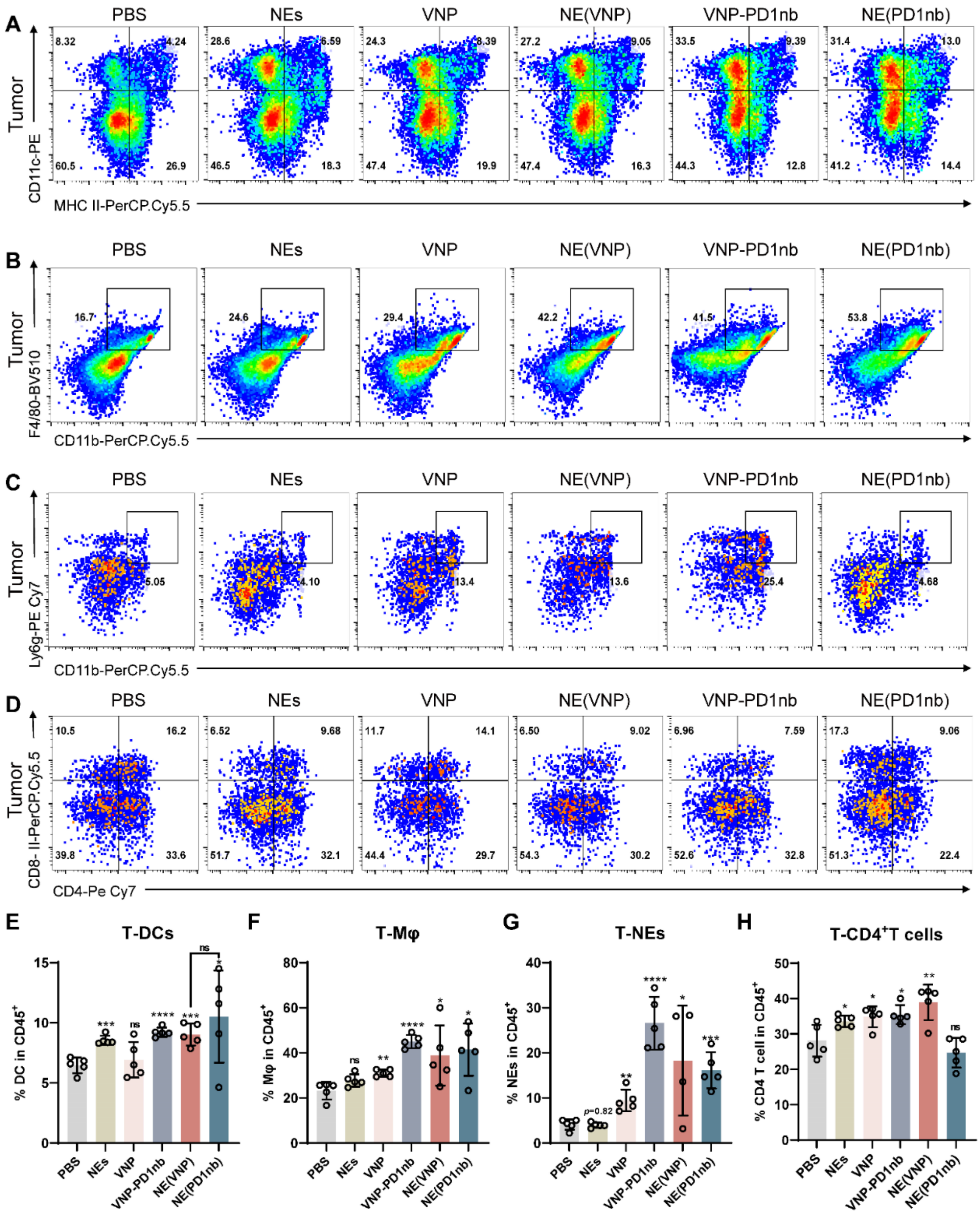

Figure S16. The change of TME after administration. A) The flow cytometry plots of tumor infiltrating DCs. B) The flow cytometry plots of tumor-infiltrating macrophages. C) The flow cytometry plots of tumor-infiltrating neutrophils. D) The flow cytometry plots of tumor-infiltrating CD4 and CD8 T-cells. E) The proportion of DCs among CD45^{+} cells in the tumor. F) Within tumor, the percentage of macrophages in CD45^{+} cells. G) Intertumoral neutrophils (% CD45^{+} cells). H) The statistic diagram of tumor infiltrating CD4 T-cells. Data are shown as the mean ± SD. **** $p < 0.0001$, *** $p < 0.001$, ** $p < 0.01$, * $p < 0.05$.

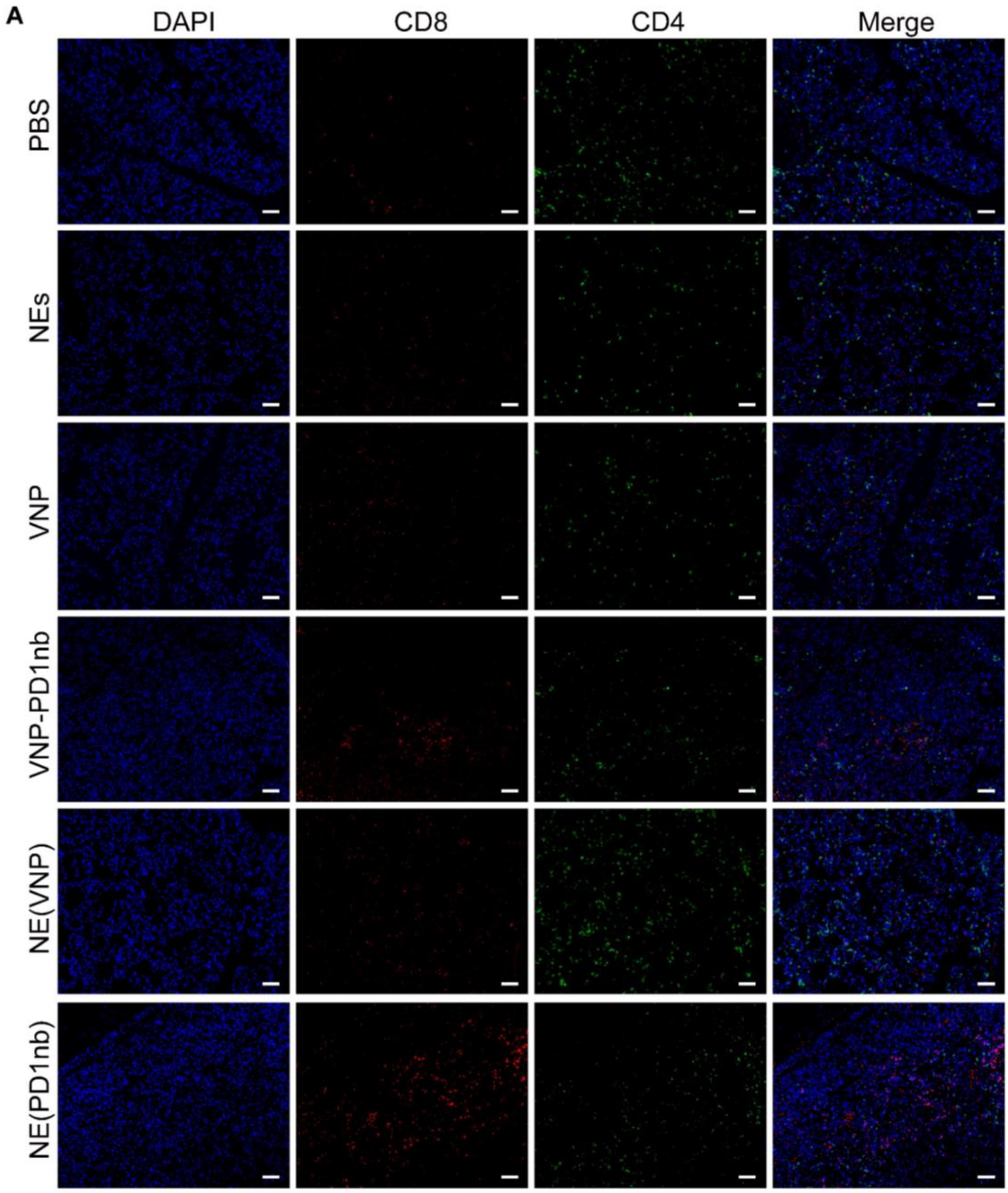

Figure. S17. A) The distribution of $CD8^+$ T-cells (Red) and $CD4^+$ T-cells (Green) in lung after administrations measured by immunofluorescence.

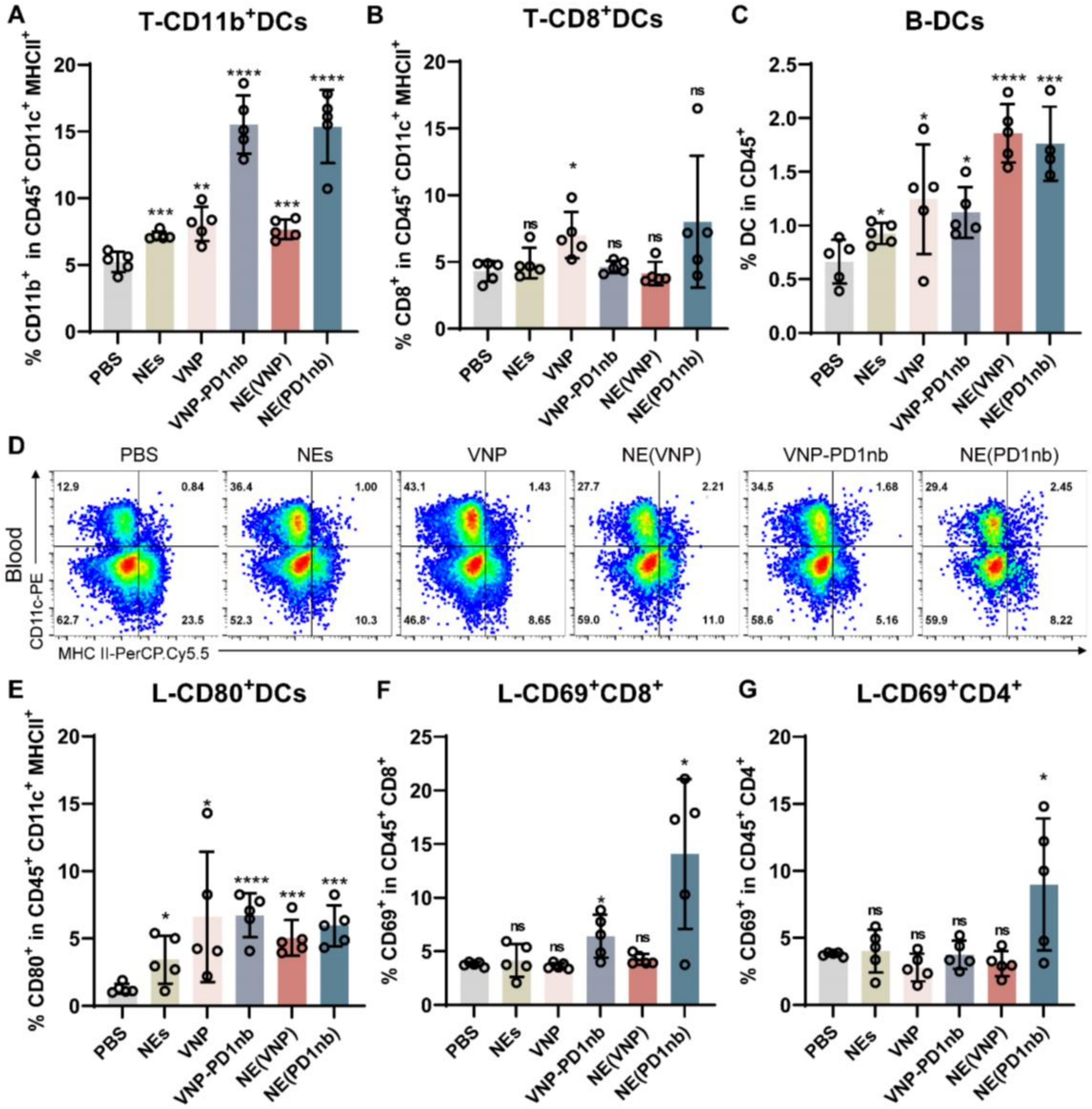

Figure S18. NE(PD1nb) induced more DCs mature and activation. A) The statistic diagram of tumor infiltrating CD11b$^+$ DCs. B) The statistic diagram of tumor infiltrating CD8$^+$ DCs. C, D) The proportion of DCs among CD45$^+$ cells in peripheral blood. E) In the TdLN, the percentage of CD80$^+$ in DCs. F) The statistic diagram of DCs in spleen. G) The statistic diagram of DCs in TdLN. Data are shown as the mean ± SD. **** $p < 0.0001$, *** $p < 0.001$, ** $p < 0.01$, * $p < 0.05$, ns: no significance.

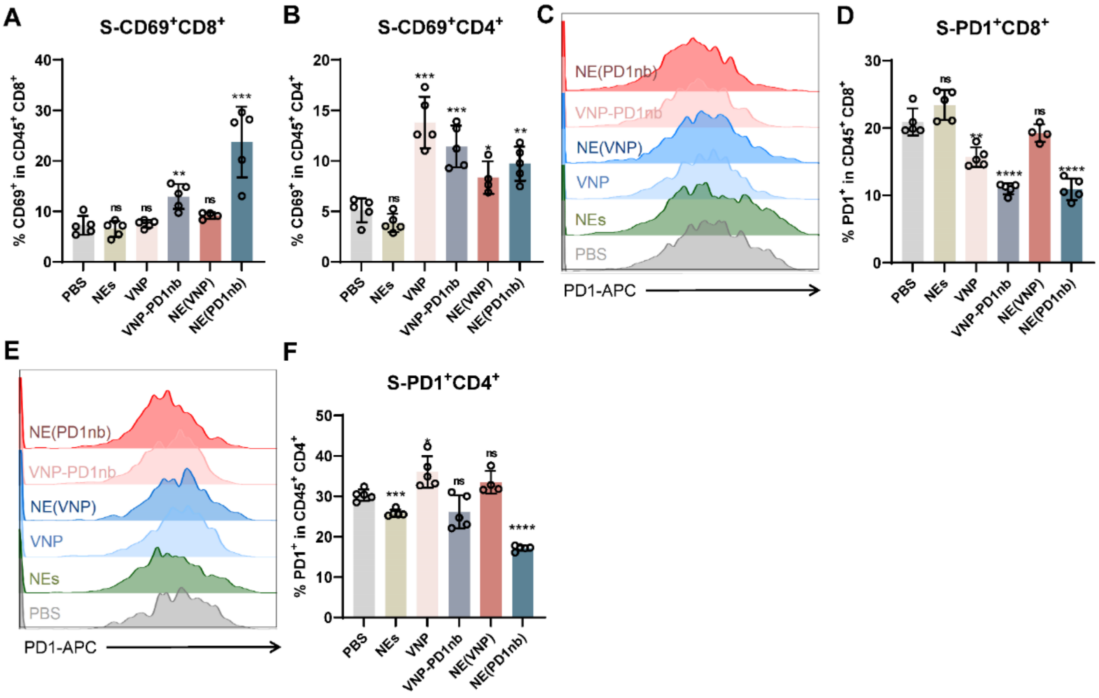

Figure S19. The change of T cells in other tumor immunity-related organs. A, B) The statistic diagram of activated CD4 and CD8 T-cells ($CD69^+$) in spleen. C, D) The percentage of $PD1^+$CD8 T-cells in spleen. E, F) The percentage of $PD1^+$CD4 T-cells in spleen. Data are shown as the mean ± SD. **** $p < 0.0001$, *** $p < 0.001$, ** $p < 0.01$, * $p < 0.05$, ns: no significance.

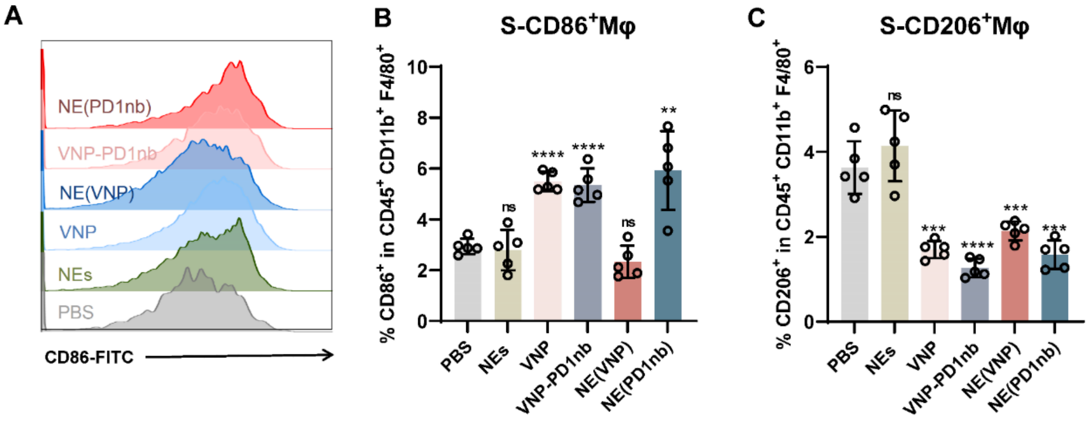

Figure S20. The state of macrophage in other tumor immunity-related organs. A-C) Within the spleen, the polarizations of macrophages were determined by FACS, (A) and (B) showed the percentage of M1-like macrophages ($CD86^+$), and the proportion of M2-like macrophages ($CD206^+$) was showed in (C). Data are shown as the mean ± SD. **** $p < 0.0001$, *** $p < 0.001$, ** $p < 0.01$, * $p < 0.05$.

Table S1. The primer sequence of RT-PCR.

Primer	Forward (5'-3')	Reverse（5'-3'）
18s	GTAACCCGTTGAACCCCATT	CCATCCAATCGGTAGTAGCG
IFN-γ	GCCACGGCACAGTCATTGA	TGCTGATGGCCTGATTGTCTT
TNF-α	ACCACGCTCTTCTGTCTACT	AGGAGGTTGACTTTCTCCTG
CCL3	ACCATGACACTCTGCAACCA	TCAGGCATTCAGTTCCAGGT
CCL2	CAGGTCCCTGTCATGCTTCT	GTCAGCACAGACCTCTCTCT
CCL5	ACCATGAAGATCTCTGCAGC	TGAACCCACTTCTTCTCTGG
ICAM1	GAAGCTTCTTTTGCTCTGCC	AGCAGTACTGGCACCAGAAT
Arg-1	GATTGGCAAGGTGATGGAAG	TCAGTCCCTGGCTTATGGTT
TGFβ-R1	GGCTTCCTGAGGAGAAGCTG	ACACTGTAATGCCTTCGCCC
TRAIL	CCTCTCGGAAAGGGCATTC	TCCTGCTCGATGACCAGCT
PDL1	CAGGACGCAGGCGTTTACTG	CTTCCCACTCACGGGTTGGT
VEGF	ATGGATGTCTACCAGCGAAG	CTGAACAAGGCTCACAGTGA
Nos2	GTTCTCAGCCCAACAATACAAGA	GTGGACGGGTCGATGTCAC
FAS	ATGCTGTGGATCTGGGCT	TCACTCCAGACATTGTCC
IL-1β	GAAATGCCACCTTTTGACAGTG	TGGATGCTCTCATCAGGACAG
IL-12	CAATCACGCTACCTCCTCTTTT	CAGCAGTGCAGGAATAATGTTTC
SIRPα	CTCTCCGCGTCCTGTTTCTG	TCTGTACCACCTAATGGGTCC
CXCR2	ATGCCCTCTATTCTGCCAGAT	GTGCTCCGGTTGTATAAGATGAC
CXCR4	GACTGGCATAGTCGGCAATG	AGAAGGGGAGTGTGATGACAAA
CXCR1	TCTGGACTAATCCTGAGGGTG	GCCTGTTGGTTATTGGAACTCTC
CCR1	CTCATGCAGCATAGGAGGCTT	ACATGGCATCACCAAAAATCCA
CCR5	TTTTCAAGGGTCAGTTCCGAC	GGAAGACCATCATGTTACCCAC
CD47	TGGTGGGAAACTACACTTGCG	CGTGCGGTTTTTCAGCTCTAT
Annexin I	GATCAAGGCCGCGTACTTACA	GCTGGAGTTTTTAGCATAGCCA

Table S2. The information of FACs antibody.

Antibodies	Article No.	Company
Ms CD45-PE Cy7	552848	BD Pharmingen™
Ms CD8a-PerCP Cy5.5	551162	BD Pharmingen™
Ms CD8-FITC	553030	BD Pharmingen™
Ms CD11c-PE	553802	BD Pharmingen™
Ms I-A/I-E BB700	746197	BD Pharmingen™
Ms CD11b- PerCP Cy5.5	550993	BD Pharmingen™
Ms CD11b- APC	557396	BD Pharmingen™
Ms CD80-BV421	562611	BD Pharmingen™
Ms CD206-AF647	565250	BD Pharmingen™
Ms CD69-BV421	562920	BD Pharmingen™
Ms F4/80-BV510	743280	BD Pharmingen™
Ms Ly6C-PE	560592	BD Pharmingen™
Ms Ly6G-PE.Cy7	560601	BD Pharmingen™
CD4-PE.Cy7	12-0043-82	eBioscience™
Granzyme B-PE	12-8898-82	eBioscience™
PD1-APC	17-9985-82	eBioscience™
TNFα-BV421	506328	Biolegend™
CD86-FITC	105005	Biolegend™
CD103-PE.Cy7	121426	Biolegend™
